# Pamiparib in combination with tislelizumab in patients with advanced solid tumours: results from the dose-expansion stage of a multicentre, open-label, phase I trial

**DOI:** 10.1038/s41416-023-02349-0

**Published:** 2023-07-20

**Authors:** Michael Friedlander, Linda Mileshkin, Janine Lombard, Sophia Frentzas, Bo Gao, Michelle Wilson, Tarek Meniawy, Sally Baron-Hay, Karen Briscoe, Nicole McCarthy, Christos Fountzilas, Andres Cervantes, Ruimin Ge, John Wu, Alexander Spira

**Affiliations:** 1 University of New South Wales Clinical School and Department of Medical Oncology, Prince of Wales Hospital, Randwick, NSW Australia; 2 Department of Medical Oncology, Peter MacCallum Cancer Centre, and the Sir Peter MacCallum Department of Oncology, The University of Melbourne, Melbourne, Parkville, VIC Australia; 3 Medical Oncology, Calvary Mater Newcastle, NSW Australia; 4 Department of Medical Oncology, Monash Health and Faculty of Medicine, Nursing and Health Sciences, Monash University, Melbourne, VIC Australia; 5 Medical Oncology Department, Blacktown Hospital, Sydney, NSW Australia; 6 Department of Cancer and Blood, Auckland City Hospital, Auckland, New Zealand; 7 Department of Medical Oncology, Linear Clinical Research and University of Western Australia, Nedlands, WA Australia; 8 Department of Medical Oncology, Royal North Shore Hospital, St Leonards, NSW Australia; 9 GenesisCare, Melbourne, VIC Australia; 10 Department of Medical Oncology, Mid North Coast Cancer Institute, Coffs Harbour, NSW Australia; 11 Department of Medical Oncology, Icon Cancer Centre Wesley, Auchenflower, QLD Australia; 12 Department of Medicine/Division of GI Medicine and Early Phase Clinical Trial Program, Roswell Park Comprehensive Cancer Center, Buffalo, NY USA; 13 Department of Medical Oncology, Hospital Clínico Universitario, INCLIVA Biomedical Research Institute, University of Valencia, Valencia, Spain; 14 Instituto de Salud Carlos III, CIBERONC, Madrid, Spain; 15 Department of Clinical Development, BeiGene (Beijing) Co., Ltd., Beijing, China; 16 Department of Biostatistics, BeiGene USA, Inc., San Mateo, CA USA; 17 Department of Medical Oncology, Virginia Cancer Specialists Research Institute, Fairfax, VA USA; 18 NEXT Oncology-Virginia, Fairfax, VA USA; 19 US Oncology Research, The Woodlands, TX USA

**Keywords:** Prostate cancer, Ovarian cancer, Small-cell lung cancer, Gastric cancer, Cancer immunotherapy

## Abstract

**Background:**

The aim of this study was to investigate the antitumour activity, safety, and tolerability of pamiparib plus tislelizumab in patients with previously treated advanced solid tumours.

**Methods:**

In this study, patients were enrolled into eight arms by tumour type. All received pamiparib 40 mg orally twice daily plus tislelizumab 200 mg intravenously every 3 weeks. The primary endpoint was objective response rate (ORR), assessed by the investigator per Response Evaluation Criteria in Solid Tumours v1.1. Secondary endpoints included duration of response (DoR), safety, and tolerability.

**Results:**

Overall, 180 patients were enrolled. In the overall population, the ORR was 20.0% (range: 0–47.4 across study arms), with median DoR of 17.1 months (95% confidence interval [CI]: 6.2, not estimable [NE]). The highest ORR was observed in the triple-negative breast cancer (TNBC) arm (patients with *BRCA*1/2 mutations and/or homologous recombination deficiency) (ORR: 47.4%; median DoR: 17.1 months [95% CI: 3.0, NE]). Treatment-emergent adverse events (TEAEs) of ≥Grade 3 occurred in 61.7% of patients. Serious TEAEs occurred in 50.0% of patients.

**Conclusions:**

Pamiparib plus tislelizumab showed a variable level of antitumour activity in patients with advanced solid tumours, with the highest ORR in TNBC and was associated with a manageable safety profile.

**Clinical trial registration:**

ClinicalTrial.gov: NCT02660034.

## Introduction

PARP 1/2 inhibitors interfere with DNA repair mechanisms by binding directly to PARP enzymes, thereby inhibiting their activity, and through trapping PARP-DNA complexes at the site of DNA damage. These effects lead to genomic instability, prevention of DNA transcription/translation, increased DNA damage, and tumour cell death [1–4]. PARP inhibitors are synthetically lethal in tumours with homologous recombination deficiencies (HRD), in particular in tumours with either a germline or somatic mutation in the breast cancer type 1/2 susceptibility gene *(BRCA*1/2*)* [5]. Several PARP inhibitors are approved for the treatment of ovarian, breast, prostate, and pancreatic cancers, including for patients with deleterious/suspected deleterious *BRCA* mutations (*BRCA*mut) and/or tumours with HRD [6–9].

PARP inhibitors may enhance the antitumour effects of immune checkpoint inhibitors, such as programmed cell death protein 1 (PD-1)/programmed death-ligand 1 (PD-L1) inhibitors [3, 4, 10]. PARP inhibitor-induced tumour cell death leads to tumour neoantigen release, facilitating potential immune responses [3, 10]. PARP inhibitors also have other effects that may potentiate antitumour immune responses and create a favourable environment for immune checkpoint blockade, such as promotion of T-cell infiltration and upregulation of interferons [3, 10, 11]. These effects may be mediated through mechanisms such as activation of the cGMP/AMP-synthase-stimulator-of-interferon genes (cGAS-STING) pathway and increased chemokine levels [3, 10]. However, PARP inhibitors also upregulate PD-L1 expression on tumour cells via various mechanisms (e.g., GSK3β, JAK2/STAT3 signalling pathway, or activation of the cGAS-STING pathway) [10–12] which could, in turn, lead to suppression of T-cell-mediated immune responses, and therefore represent a mechanism of resistance to PARP inhibitors [10, 12].

Preclinical studies suggest addition of a PD-(L)1 inhibitor can resensitise cells treated with PARP inhibitors to T-cell cytotoxicity, which restores the reduced antitumour immunity caused by upregulation of PD-L1 expression and enhances antitumour activity [10–12]. Furthermore, HRD tumours have been reported to exhibit traits that may favour immune checkpoint blockade, such as high neoantigen load, increased PD-L1 expression, and increased levels of tumour-infiltrating lymphocytes [13]. Consequently, several clinical trials have been initiated to investigate the effects of combination PARP inhibitors with PD-(L)1 inhibitors in various solid tumours [3, 10, 14–19].

Pamiparib (developed by BeiGene, Ltd.) is a potent, selective, investigational small molecule inhibitor of PARP1 and PARP2 that has demonstrated brain penetration and PARP-DNA complex trapping in preclinical studies [20, 21]. In phase I and II clinical studies, pamiparib demonstrated antitumour activity and induced durable responses in patients with epithelial ovarian cancer (EOC) and HER2-negative (HER2−) breast cancer with germline *BRCA*mut [22–25].

Several PD-(L)1 inhibitors are approved as monotherapy and/or in combination with chemotherapy and/or other agents for the treatment of a range of solid tumours, with survival benefits demonstrated versus placebo and various other comparators [26]. Tislelizumab is an anti-PD-1 monoclonal antibody with high affinity and binding specificity for PD-1 [27, 28] and was specifically engineered to minimise Fc-gamma receptor binding on macrophages [28, 29]. Clinical studies have demonstrated durable antitumour efficacy of tislelizumab in various solid tumours [30–33], and it is approved in China for the treatment of several tumour types.

A phase Ia/b study was initiated to investigate the combination of pamiparib with tislelizumab in patients with advanced solid tumours [34]. The study comprised two phases: dose escalation (part A) and dose expansion (part B). In the dose-escalation phase, the combination was generally well tolerated and demonstrated antitumour activity, supporting its continued investigation in the dose-expansion phase [34]. The objective response rate (ORR) was 20.4%, with responses seen in patients with gynaecological cancers (ovarian, fallopian or peritoneal) and breast cancer [34]. The recommended phase II dose was pamiparib 40 mg orally twice daily plus tislelizumab 200 mg intravenously (IV) every 3 weeks (Q3W) [34]. Here, we report results from the dose-expansion phase, which sought to investigate the antitumour activity, safety, and tolerability of pamiparib combined with tislelizumab at the recommended phase II dose in patients with a variety of advanced solid tumour types (with and without a germline or somatic *BRCA*mut and HRD), including cohorts of patients with EOC and TNBC.

## Methods

### Study design

Part B was a multicentre, open-label, multiple-arm, dose-expansion study that evaluated the antitumour activity, safety, and tolerability of pamiparib plus tislelizumab (NCT02660034). The study enrolled patients into eight arms according to tumour type (Supplementary Fig. S1). The study arms included patients with ovarian, TNBC, prostate, small cell lung, gastric or gastroesophageal junction, urothelial, or pancreatic cancers, and an exploratory arm included patients with non-ovarian gynaecological cancers, and patients with tumours that were mismatch repair deficient or HRD who were not eligible for inclusion in other arms. Patients were not randomised and there was no blinding of study treatments. Study endpoints were evaluated independently in each arm to explore the clinical activity, safety, and tolerability of pamiparib plus tislelizumab in each selected tumour type. All patients provided written informed consent before study participation. All relevant Institutional Review Boards/Independent Ethics Committees approved the study, which was carried out in accordance with the International Conference on Harmonization Good Clinical Practice Guideline, the principles of the Declaration of Helsinki, and local laws and regulations.

### Participants

Eligible patients were adults (≥18 years of age) with histologically confirmed malignancies that have progressed to the advanced/metastatic stage, with measurable disease per Response Evaluation Criteria in Solid Tumours (RECIST) v1.1, and an Eastern Cooperative Oncology Group (ECOG) performance status of 0 or 1.

As outlined in Supplementary Fig. S1, tumour-specific eligibility criteria per study arm were as follows: relapsed, platinum-sensitive high-grade epithelial, non-mucinous, ovarian cancer, fallopian tube, or primary peritoneal cancer (termed “EOC” hereafter), with a known germline or somatic *BRCA*mut and/or HRD (Arm 1a), or without mutation (*BRCA*wt) and with homologous recombination proficiency (HRP) (Arm 1b); TNBC with either a known germline or somatic *BRCA*mut and/or with documented HRD (Arm 2); metastatic castration-resistant prostate cancer (mCRPC) with either a known germline or somatic *BRCA*mut and/or with documented HRD (Arm 3); extensive-stage small cell lung cancer (SCLC) (Arm 4); HER2− gastric or gastroesophageal junction cancer (Arm 5); locally advanced or metastatic urothelial cancer (Arm 6); advanced or metastatic pancreatic adenocarcinoma (Arm 7). The final arm of the study (Arm 8) enrolled patients with advanced or metastatic recurrent non-ovarian gynaecological cancers (endometrial cancer or cancer of the cervix), and patients with tumours known to be either mismatch repair deficient or HRD that were not eligible for inclusion in any other arms of the trial but may be expected to benefit from the combination of a PARP inhibitor and a PD-1 inhibitor (termed “exploratory arm” hereafter). For the purposes of determination of study eligibility, patients’ *BRCA*mut and HRD/HRP status in Arms 1–3 were determined using historical clinical test results or, when results were not available, based on central testing of samples using the Myriad BRCAnalysis CDx or Myriad myChoice^®^ CDx methods (for a germline *BRCA*mut and HRD/HRP status, respectively). For patients enrolled on the basis of local historical clinical test results, confirmatory central testing was subsequently performed using archival or fresh samples, where possible.

Prior treatment varied by tumour type, but all patients were required to have received standard of care for the treatment of their disease (Supplementary Fig. S1). Patients who had received treatment in the advanced/metastatic setting were also eligible. If received, chemotherapy or investigational therapy must have been completed ≥4 weeks (or ≥5 half-lives, whichever was longer) prior to administration of the study treatment, and palliative radiotherapy must have been completed ≥2 weeks prior.

Key exclusion criteria were platinum-resistant/refractory EOC and prior treatment with therapies targeting PD-1, PD-L1, or PARP. Full eligibility criteria are provided in the Supplementary Appendix.

### Interventions

All patients received pamiparib 40 mg orally twice daily plus tislelizumab 200 mg IV Q3W (Supplementary Fig. S1). The dosing regimen was selected based on the previously reported results of the dose-escalation phase of the study [34]. The initial infusion of tislelizumab was administered over 60 min and subsequently reduced to 30 min if well tolerated. Study treatments were administered until disease progression, unacceptable toxicity, loss to follow-up, death, or discontinuation for other reasons. Continued treatment beyond progression was permitted if “pseudo-progression” was suspected by the investigator, provided protocol-specified criteria were met.

If required to manage adverse events (AEs), pamiparib dose reduction to 20 mg was permitted, and dosing could also be withheld for up to 28 days consecutively. No dose reduction was permitted for tislelizumab, but dose delays of less than 12 weeks were permitted. Whether an AE was possibly related to either pamiparib or tislelizumab alone, or both, was assessed by the investigator, and the study drug(s) considered to be responsible were modified accordingly.

### Endpoints and assessments

The primary endpoint of this study was ORR, defined as the proportion of patients with a documented complete response (CR) or partial response (PR) per RECIST v1.1, as assessed by investigators. Tumour imaging was performed within 28 days prior to enrolment, every 9 weeks (±1 week) in the first 12 months of enrolment, and every 12 weeks (±1 week) thereafter. Computed tomography (CT) or magnetic resonance imaging techniques were used, with a preference for CT. The same imaging technique was used throughout the study for each individual patient. All known disease was documented at baseline as target or non-target lesions per RECIST v1.1.

Secondary endpoints included: progression-free survival (PFS), duration of response (DoR), disease control rate (DCR), and clinical benefit rate (CBR), all by investigator per RECIST v1.1; and overall survival (OS), safety and tolerability, pharmacokinetic parameters, and the immunogenicity of tislelizumab. Safety and tolerability were assessed by the incidence and nature of AEs. AEs were coded using the Medical Dictionary for Regulatory Activities (MedDRA) version 22.0 and graded per National Cancer Institute’s Common Terminology Criteria for Adverse Events version 4.03. The assignment of immune-mediated AEs was assessed by investigators based on diagnostic test results and clinical judgement, and the exclusion of alternative explanations, in line with criteria defined in the study protocol. Other safety assessments included vital signs, electrocardiograms, laboratory analyses, and physical and ophthalmologic examinations. In addition, as an elevated incidence of hepatic AEs was observed with the combination of pamiparib and tislelizumab in the dose-escalation part of the study [34], the incidence of hepatic AEs is reported. Pharmacokinetic parameters assessed included C_max_, C_trough_ and T_max_ for pamiparib and C_trough_ for tislelizumab. Immunogenic responses to tislelizumab were assessed in terms of the incidence of anti-drug antibodies (ADAs).

### Statistical analyses

This dose-expansion study planned to initially enrol 20 patients in each tumour-specific arm. The probability of observing at least one responder was calculated to be approximately 88% in each dose-expansion arm (*n* = 20) if the underlying ORR was as low as 10%. Twenty additional patients could be enrolled in any arm to further evaluate antitumour activity if evidence of activity was observed. Since a precise estimate of ORR is difficult to predict due to the heterogeneity of patients enrolled within each arm, the Bayesian predictive probability, which evaluates the statistical strength of the pamiparib plus tislelizumab combination regimen versus the standard chemotherapy, will be used to provide guidance to decide whether to enrol an additional twenty patients. For example, for an arm with a historical response rate of 10%, at least two responders of the initial 20 patients should be observed to have a predictive probability of >10% superiority over the 10% historical rate in a total of 50 patients; however, for an arm with higher expected response rate (e.g., >30% ORR in arm 1a), at least six responders are required to have a >10% predictive probability in order to expand the arm beyond the initial 20 patients. Conversely, a decision could be made to stop enrolment in an arm early due to suboptimal clinical antitumour activity. Efficacy and safety analyses were performed in the safety analysis set, which included all patients who received any dose of any study treatment. Pharmacokinetic analyses were performed in all patients with valid pharmacokinetic sampling after treatment with study drug(s) (the pharmacokinetic analysis set). Immunogenicity analyses were performed among patients with evaluable data (ADA evaluable population).

Data for each arm in this study were analysed independently. For PFS and OS, median durations and event-free rates at various timepoints were estimated using the Kaplan–Meier method: for medians and other quartiles, 95% confidence intervals (CIs) were estimated using the Brookmeyer and Crowley method; for event-free rates, 95% CIs were estimated using the Kaplan–Meier method and the Greenwood formula. Patients who remained alive before data cutoff or discontinuation of the study were censored at the last date the subject was known to be alive. DoR analyses only included patients who responded to treatment, with the same censoring rules used for PFS, and Kaplan–Meier curves were used to estimate median DoR and 95% CIs. The incidence of treatment-emergent AEs (TEAEs), incidence of ADAs, laboratory test results, vital signs, pharmacokinetic parameters, and their changes from baseline were summarised using descriptive statistics. All safety analyses were performed by arms and by the total combination of cohorts in the safety analysis set. All calculations and analyses were conducted using SAS version 9.2 or higher. The study was not powered to detect statistical significance.

## Results

### Patients and treatment

Patients were recruited from 25 sites across five countries (Australia, France, New Zealand, Spain, and the United States) between 21 July 2017 and 9 April 2019, with a median time of 25.8 months from initial diagnosis to study entry. In total, 180 patients were assigned to the eight study arms. Patient demographics and baseline characteristics, including *BRCA*mut/HRD status, are presented in Table 1 and Supplementary Table S1. All 180 patients were included in the safety analysis set, of whom all except two patients had received at least one prior line of systemic therapy.

**Table 1:** Patient demographics and baseline characteristics.

	EOC *BRCA*mut and/or HRD (Arm 1a; *n* = 23)	EOC *BRCA*wt and HRP (Arm 1b; *n* = 23)	TNBC *BRCA*mut and/or HRD (Arm 2; *n* = 19)	mCRPC *BRCA*mut and/or HRD (Arm 3; *n* = 20)	SCLC (Arm 4; *n* = 23)	HER2− G/GEJ cancer (Arm 5; *n* = 20)	Urothelial cancer (Arm 6; *n* = 21)	Pancreatic cancer (Arm 7; *n* = 21)	Exploratory arm^a^ (Arm 8; *n* = 10)	Total (*N* = 180)
Median age (years)	59.0	73.0	49.0	68.0	65.0	62.0	70.0	64.0	62.5	64.0
Sex, *n* (%)										
Male	0 (0.0)	0 (0.0)	0 (0.0)	20 (100.0)	13 (56.5)	17 (85.0)	15 (71.4)	12 (57.1)	5 (50.0)	82 (45.6)
Female	23 (100.0)	23 (100.0)	19 (100.0)	0 (0.0)	10 (43.5)	3 (15.0)	6 (28.6)	9 (42.9)	5 (50.0)	98 (54.4)
Race, *n* (%)										
Asian	2 (8.7)	0 (0.0)	0 (0.0)	0 (0.0)	2 (8.7)	1 (5.0)	0 (0.0)	0 (0.0)	2 (20.0)	7 (3.9)
Black or African American	0 (0.0)	0 (0.0)	0 (0.0)	0 (0.0)	0 (0.0)	1 (5.0)	0 (0.0)	2 (9.5)	0 (0.0)	3 (1.7)
Native Hawaiian or other Pacific Islander	1 (4.3)	0 (0.0)	0 (0.0)	1 (5.0)	1 (4.3)	0 (0.0)	0 (0.0)	0 (0.0)	0 (0.0)	3 (1.7)
White	20 (87.0)	23 (100.0)	17 (89.5)	18 (90.0)	18 (78.3)	16 (80.0)	18 (85.7)	19 (90.5)	8 (80.0)	157 (87.2)
Unknown	0 (0.0)	0 (0.0)	1 (5.3)	1 (5.0)	1 (4.3)	0 (0.0)	1 (4.8)	0 (0.0)	0 (0.0)	4 (2.2)
Not reported	0 (0.0)	0 (0.0)	1 (5.3)	0 (0.0)	0 (0.0)	0 (0.0)	1 (4.8)	0 (0.0)	0 (0.0)	2 (1.1)
Missing	0 (0.0)	0 (0.0)	0 (0.0)	0 (0.0)	1 (4.3)	2 (10.0)	1 (4.8)	0 (0.0)	0 (0.0)	4 (2.2)
ECOG PS, *n* (%)										
0	10 (43.5)	11 (47.8)	12 (63.2)	7 (35.0)	6 (26.1)	11 (55.0)	6 (28.6)	10 (47.6)	5 (50.0)	78 (43.3)
1	13 (56.5)	12 (52.2)	7 (36.8)	13 (65.0)	17 (73.9)	9 (45.0)	15 (71.4)	11 (52.4)	5 (50.0)	102 (56.7)
Homologous recombination status,^b^ *n* (%)										
HRD	22 (95.7)	0 (0.0)	13 (68.4)	16 (80.0)	NA	NA	NA	NA	NA	51 (28.3)
HRP	1 (4.3)^c^	17 (73.9)	0 (0.0)	0 (0.0)	NA	NA	NA	NA	NA	18 (10.0)
Not centrally assessed	0 (0.0)	6 (26.1)^d^	6 (31.6)^e^	4 (20.0)^f^	NA	NA	NA	NA	NA	16 (8.9)
Germline *BRCA* mutation,^b^ *n* (%)										
Mutant	4 (17.4)	0 (0.0)	10 (52.6)	0 (0.0)	NA	NA	NA	NA	NA	14 (7.8)
Wild type	14 (60.9)	20 (87.0)	5 (26.3)	2 (10.0)	NA	NA	NA	NA	NA	41 (22.8)
Not centrally assessed	5 (21.7)	3 (13.0)	4 (21.1)	18 (90.0)	NA	NA	NA	NA	NA	30 (16.7)
Number of lines of prior systemic therapies, *n* (%)										
0	0 (0.0)	1 (4.3)	0 (0.0)	0 (0.0)	0 (0.0)	1 (5.0)	0 (0.0)	0 (0.0)	0 (0.0)	2 (1.1)
1	3 (13.0)	1 (4.3)	10 (52.6)	1 (5.0)	16 (69.6)	11 (55.0)	14 (66.7)	3 (14.3)	3 (30.0)	62 (34.4)
2	6 (26.1)	8 (34.8)	2 (10.5)	5 (25.0)	6 (26.1)	8 (40.0)	6 (28.6)	13 (61.9)	3 (30.0)	57 (31.7)
≥3	14 (60.9)	13 (56.5)	7 (36.8)	14 (70.0)	1 (4.3)	0 (0.0)	1 (4.8)	5 (23.8)	4 (40.0)	59 (32.8)

Additional baseline characteristics are reported in Supplementary Table S1.

*BRCAmut* breast cancer type 1/2 susceptibility gene mutation, *BRCAwt* breast cancer type 1/2 susceptibility gene wildtype, *ECOG PS* Eastern Cooperative Oncology Group performance status, *EOC* epithelial ovarian cancer, *G/GEJ* gastric or gastroesophageal junction, *HER2−* HER2 negative, *HRD* homologous recombination deficiency, *HRP* homologous recombination proficiency, *mCRPC* metastatic castration-resistant prostate cancer, *NA* not assessed, *PD-1* programmed cell death protein 1, *SCLC* small cell lung cancer, *TNBC* triple-negative breast cancer.

^a^Patients with non-ovarian gynaecological cancers (endometrial cancer or cancer of the cervix) and patients with tumours known to be mismatch repair deficient or HRD that are not eligible for inclusion in any other arms of the trial but that may be expected to benefit from the PARP/PD-1 inhibitor combination (see Supplementary Table S1 for the full list of cancer types enrolled in this arm).

^b^By central laboratory analysis. (41 patients had known *BRCA* mutation status based on both historical and central tests; 13 patients had results for both historical and central HRD tests. Concordance between historical and central test results was seen in 38/41 *BRCA* test results and 10/13 HRD test results).

^c^This patient was enrolled on the basis of a historical status of HRD, which was not confirmed by a later central laboratory HRD test (central assessment reported HRP).

^d^These patients either did not have central HRD assessment, or had inconclusive results; one patient had a historical HRP status, and all six had a germline *BRCA*wt status per central assessment.

^e^These patients did not have central HRD assessment, and were enrolled on the basis of a historical germline *BRCA*mut status. Among these six patients, four had a centrally confirmed germline *BRCA*mut status, and two were not confirmed by a later central germline *BRCA*mut assessment (central assessment reported germline *BRCA*wt status).

^f^These patients either did not have central HRD assessment, or had inconclusive results; three patients had a historical status of HRD; one had unknown historical HRD status and was germline *BRCA*wt based on historical assessment (no central assessment performed).

At the data cutoff of 25 September 2020, all 180 patients (100.0%) had discontinued treatment with pamiparib and tislelizumab, and had also discontinued from the study (Supplementary Table S2). Progressive disease (PD) was the most common reason for treatment discontinuation for both pamiparib (73.3% of patients) and tislelizumab (71.7% of patients). Following completion or discontinuation of treatment, patients were followed for their survival status until discontinuation from the study due to patient death or other reasons. In total, 68.9% of patients discontinued the study due to death, 3.3% due to withdrawal by patient, 1.7% due to loss to follow-up, 0.6% due to physician decision, 0.6% due to study termination by the sponsor, and 25.0% due to other reasons, including transfer to a long-term extension study of tislelizumab and/or pamiparib (NCT04164199).

The median duration of exposure to tislelizumab was 104.0 days (range: 21–994 days), and the median number of completed cycles was 5 (range: 1–46 cycles). The median duration of exposure to pamiparib was 104.0 days (range: 6–952 days), the median relative dose intensity was 98% (range: 24–100%), and the median number of completed cycles was 5 (range: 1–46 cycles).

### Antitumour activity

**Fig. 1:**
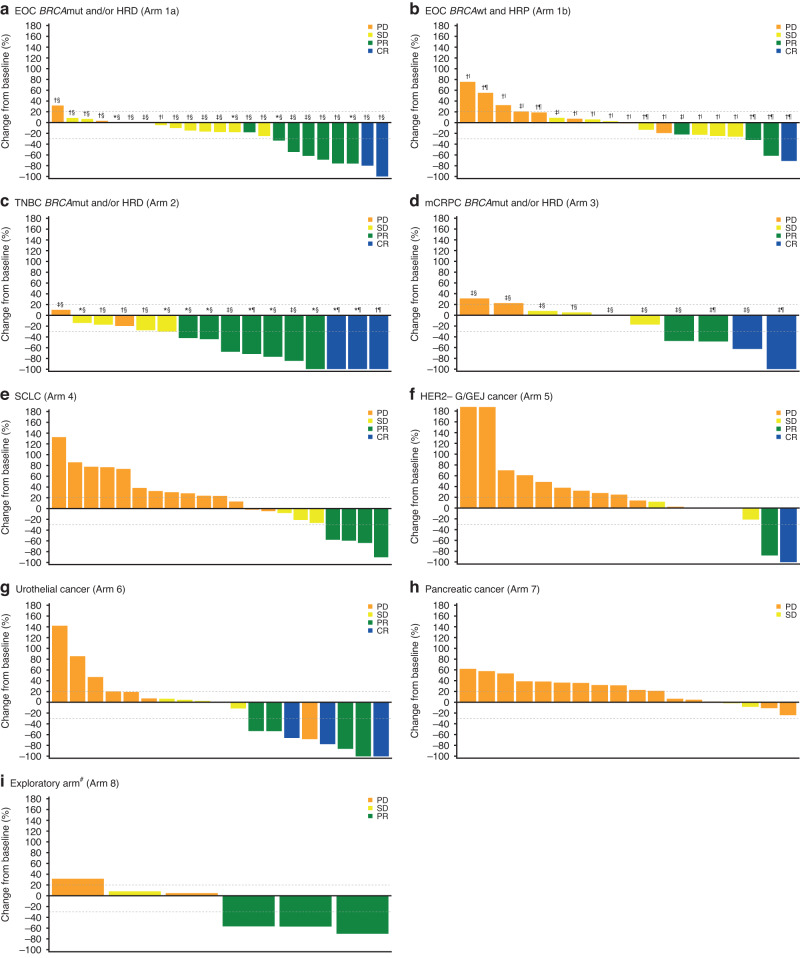
Maximum reduction from baseline in target lesion diameter per study arm. *BRCAmut by central testing; ^†^*BRCA*wt by central testing; ^‡^*BRCA* unknown by central testing; ^§^HRD by central testing; ^|^HRP by central testing; ^¶^HRD unknown by central testing. ^#^Patients with non-ovarian gynaecological cancers (endometrial cancer or cancer of the cervix) and patients with tumours known to be mismatch repair deficient or HRD that are not eligible for inclusion in any other arms of the trial but that may be expected to benefit from the PARP/PD-1 inhibitor combination (see Supplementary Table S1 for the full list of cancer types enrolled in this arm). Dotted lines at −30% and 20% indicate the boundaries for disease response and progression, respectively. Data are presented only for patients with an evaluable post-baseline assessment of target lesions. Data cutoff: 25 September 2020. *BRCA*mut breast cancer type 1/2 susceptibility gene mutation, *BRCA*wt breast cancer type 1/2 susceptibility gene wildtype, CR complete response, EOC epithelial ovarian cancer, G/GEJ gastric or gastroesophageal junction, HER2− HER2 negative, HRD homologous recombination deficiency, HRP homologous recombination proficiency, mCRPC metastatic castration-resistant prostate cancer, PD-1 programmed cell death protein 1, PD progressive disease, PR partial response, SCLC small cell lung cancer, SD stable disease, TNBC triple-negative breast cancer.

ORR per RECIST v1.1 in the overall study population was 20.0%, with responses occurring in 36 patients in total (Table 2). ORR ranged from 0% to 47.4% across study arms. Overall, 12 (6.7%) patients achieved a CR and 24 (13.3%) patients achieved a PR, while stable disease was observed in 50 (27.8%) patients (Table 2). The highest ORR was observed in patients with TNBC (Arm 2; 13 out of the 19 patients had centrally confirmed HRD, of whom six also had a centrally confirmed germline *BRCA*mut). In this arm, the ORR was 47.4% (nine of 19 patients), with three patients achieving a CR (15.8%; two had a centrally confirmed germline *BRCA*mut and one had centrally confirmed germline *BRCA*wt). Patients with EOC with *BRCA*mut and/or HRD had an ORR of 30.4% (seven of 23 patients), with a CR rate of 8.7% (two of 23 patients; both patients had centrally confirmed HRD and germline *BRCA*wt). The change in target lesion size from baseline in each study arm is shown in Fig. 1.

**Table 2:** Tumour response per RECIST v1.1, progression-free survival and overall survival.

	EOC *BRCA*mut and/or HRD (Arm 1a; *n* = 23)	EOC *BRCA*wt and HRP (Arm 1b; *n* = 23)	TNBC *BRCA*mut and/or HRD (Arm 2; *n* = 19)	mCRPC *BRCA*mut and/or HRD (Arm 3; *n* = 20)	SCLC (Arm 4; *n* = 23)	HER2− G/GEJ cancer (Arm 5; *n* = 20)	Urothelial cancer (Arm 6; *n* = 21)	Pancreatic cancer (Arm 7; *n* = 21)	Exploratory arm^a^ (Arm 8; *n* = 10)	Total (*N* = 180)
ORR, *n* (%)	7 (30.4)	3 (13.0)	9 (47.4)	4 (20.0)	2 (8.7)	2 (10.0)	6 (28.6)	0 (0.0)	3 (30.0)	36 (20.0)
Best overall response, *n* (%)^b^										
CR	2 (8.7)	1 (4.3)	3 (15.8)	2 (10.0)	0 (0.0)	1 (5.0)	3 (14.3)	0 (0.0)	0 (0.0)	12 (6.7)
PR	5 (21.7)	2 (8.7)	6 (31.6)	2 (10.0)	2 (8.7)	1 (5.0)	3 (14.3)	0 (0.0)	3 (30.0)	24 (13.3)
SD	14 (60.9)	8 (34.8)	5 (26.3)	4 (20.0)	5 (21.7)	5 (25.0)	6 (28.6)	2 (9.5)	1 (10.0)	50 (27.8)
Non-CR/ non-PD	0 (0.0)	0 (0.0)	0 (0.0)	7 (35.0)	0 (0.0)	0 (0.0)	0 (0.0)	0 (0.0)	0 (0.0)	7 (3.9)
PD	2 (8.7)	8 (34.8)	2 (10.5)	3 (15.0)	14 (60.9)	12 (60.0)	7 (33.3)	16 (76.2)	2 (20.0)	66 (36.7)
NE	0 (0.0)	1 (4.3)	2 (10.5)	1 (5.0)	0 (0.0)	0 (0.0)	0 (0.0)	0 (0.0)	0 (0.0)	4 (2.2)
DCR, *n* (%)	21 (91.3)	11 (47.8)	14 (73.7)	15 (75.0)	7 (30.4)	7 (35.0)	12 (57.1)	2 (9.5)	4 (40.0)	93 (51.7)
CBR, *n* (%)	15 (65.2)	7 (30.4)	11 (57.9)	10 (50.0)	4 (17.4)	4 (20.0)	8 (38.1)	1 (4.8)	3 (30.0)	63 (35.0)
Median DoR, months (95% CI)	11.2 (6.2, NE)	6.2 (3.8, NE)	17.1 (3.0, NE)	NR (4.1, NE)	6.2 (4.3, 8.1)	NR (NE, NE)	NR (5.7, NE)	–	NR (22.4, NE)	17.1 (6.2, NE)
Median PFS, months (95% CI)	8.2 (5.2, 11.8)	3.5 (1.9, 7.6)	8.4 (3.9, 19.0)	10.4 (4.3, 16.2)	2.0 (1.7, 2.3)	2.1 (1.9, 4.1)	3.5 (1.9, 7.5)	1.9 (1.1, 2.1)	2.2 (1.2, 24.4)	4.0 (2.2, 5.2)
Median follow-up time for OS, months (95% CI)	27.4 (18.3, 29.0)	25.2 (12.5, 30.4)	18.7 (15.9, 21.4)	21.2 (19.1, 24.4)	29.4 (8.7, 29.4)	28.3 (19.3, 30.2)	23.3 (19.8, 26.0)	27.4 (NE, NE)	31.0 (30.3, 31.6)	23.3 (20.7, 25.6)
Median OS, months (95% CI)	20.9 (13.5, NE)	18.7 (6.1, 27.0)	15.8 (10.4, NE)	21.2 (10.5, NE)	6.9 (3.3, 11.5)	7.4 (3.3, 13.4)	8.4 (4.4, 17.1)	4.1 (2.9, 5.0)	4.1 (1.2, 19.5)	10.4 (7.7, 14.2)

Event-free rates for PFS and OS at various time points are reported in Supplementary Table S3. Tumour responses were assessed by investigators per RECIST v1.1. ORR was defined as the proportion of patients with a documented CR or PR. DCR was defined as the proportion of patients whose best overall response was CR, PR, or SD. CBR was defined as the proportion of patients who had a CR, PR, or SD of at least 24 weeks in duration. DoR was defined as the time from the first determination of an objective response, until the first documentation of progression or death, whichever occurred first. PFS was defined as the time between receiving the first dose of the study drug and the first determination of objectively documented tumour progression, or death, whichever occurred first. OS was defined as the time between receiving the first dose of the study drug and death due to any cause. For PFS and OS, medians were estimated by the Kaplan–Meier method with 95% CIs estimated using the method of Brookmeyer and Crowley. Data cutoff: 25 September 2020.

*BRCAmut* breast cancer type 1/2 susceptibility gene mutation, *BRCAwt* breast cancer type 1/2 susceptibility gene wildtype, *CBR* clinical benefit rate, *CI* confidence interval, *CR* complete response, *DCR* disease control rate, *DoR* duration of response, *EOC* epithelial ovarian cancer, *G/GEJ* gastric or gastroesophageal junction, *HER2*− HER2 negative, *HRD* homologous recombination deficiency, *HRP* homologous recombination proficiency, *mCRPC* metastatic castration-resistant prostate cancer, *NE* not estimable, *NR* not reached, *ORR* objective response rate, *OS* overall survival, *PD* progressive disease, *PD-1* programmed cell death protein 1, *PFS* progression-free survival, *PR* partial response, *RECIST v1.1* Response Evaluation Criteria in Solid Tumours version 1.1, *SCLC* small cell lung cancer, *SD* stable disease, *TNBC* triple-negative breast cancer.

^a^Patients with non-ovarian gynaecological cancers (endometrial cancer or cancer of the cervix) and patients with tumours known to be mismatch repair deficient or HRD that are not eligible for inclusion in any other arms of the trial but that may be expected to benefit from the PARP/PD-1 inhibitor combination (see Supplementary Table S1 for the full list of cancer types enrolled in this arm).

^b^Of the 180 patients, 163 patients had both baseline and post-baseline tumour assessments.

In the overall population, median DoR among responders was 17.1 months (95% CI: 6.2, not estimable [NE]) (Table 2). Where reached, median DoR ranged from 6.2 months in both EOC with *BRCA*wt and HRP (95% CI: 3.8, NE), and SCLC (95% CI: 4.3, 8.1), to 17.1 months (95% CI: 3.0, NE) in TNBC. Median DoR was 11.2 months (95% CI: 6.2, NE) in EOC with *BRCA*mut and/or HRD, but was not reached by responders in the remaining arms. In the overall population, DCR was 51.7% and CBR was 35.0%.

**Fig. 2:**
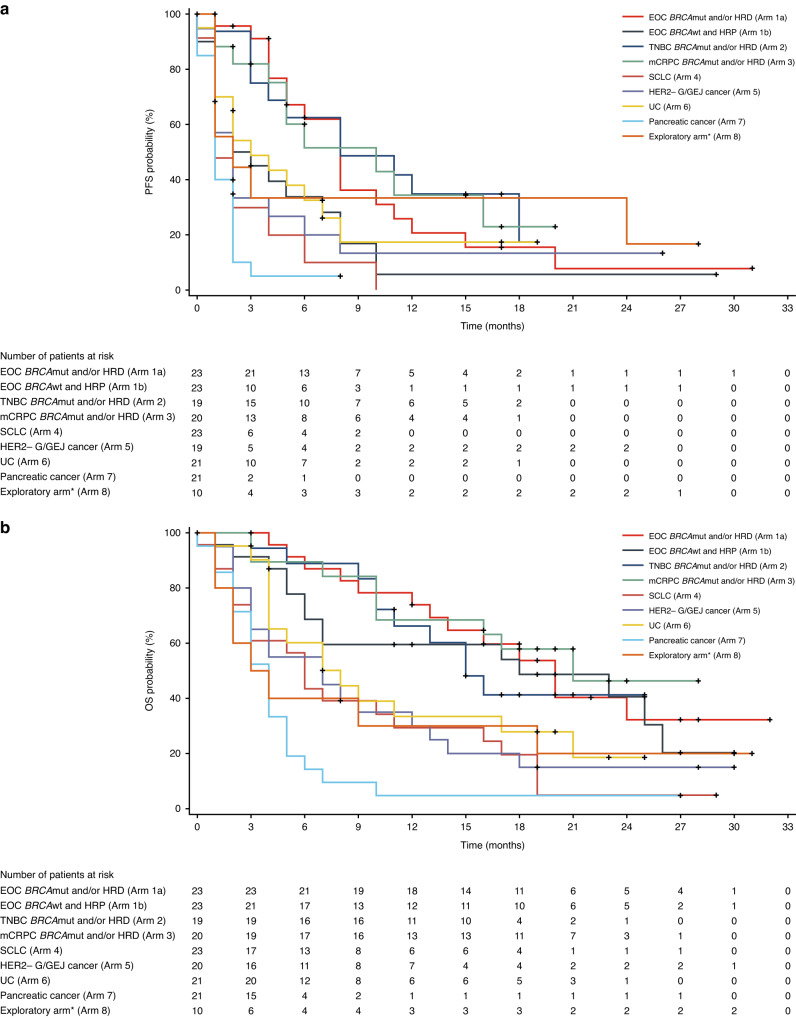
Progression-free survival and overall survival. **a** Progression-free survival. **b** Overall survival. *Patients with non-ovarian gynaecological cancers (endometrial cancer or cancer of the cervix) and patients with tumours known to be mismatch repair deficient or HRD that are not eligible for inclusion in any other arms of the trial but that may be expected to benefit from the PARP/PD-1 inhibitor combination (see Supplementary Table S1 for the full list of cancer types enrolled in this arm). Data cutoff: 25 September 2020. *BRCA*mut breast cancer type 1/2 susceptibility gene mutation, *BRCA*wt breast cancer type 1/2 susceptibility gene wildtype, EOC epithelial ovarian cancer, G/GEJ gastric or gastroesophageal junction, HER2− HER2 negative, HRD homologous recombination deficiency, HRP homologous recombination proficiency, mCRPC metastatic castration-resistant prostate cancer, OS overall survival, PD-1 programmed cell death protein 1, PFS progression-free survival, SCLC small cell lung cancer, TNBC triple-negative breast cancer.

Median PFS and OS were 4.0 months (95% CI: 2.2, 5.2) and 10.4 months (95% CI: 7.7, 14.2), respectively (Table 2). The longest PFS was seen in the arms including patients with mCRPC, TNBC, and EOC with *BRCA*mut and/or HRD, with observed median PFS of 10.4 (95% CI: 4.3, 16.2), 8.4 (95% CI: 3.9, 19.0), and 8.2 (95% CI: 5.2, 11.8) months, respectively. Median OS in these arms was 21.2 months (95% CI: 10.5, NE) in mCRPC, 20.9 months (95% CI: 13.5, NE) in EOC with *BRCA*mut and/or HRD, and 15.8 months (95% CI: 10.4, NE) in TNBC. PFS and OS rates at various timepoints are reported in Supplementary Table S3. Kaplan–Meier curves for PFS and OS are presented for all arms in Fig. 2, and are also presented discretely for the TNBC arm in Supplementary Fig. S2.

### Safety and tolerability

In the overall study population, all patients except one (99.4%) experienced at least one TEAE (Table 3). TEAEs were defined using MedDRA preferred terms. Nausea was the most commonly reported any grade TEAE (56.1% of patients) (Table 4). TEAEs of ≥Grade 3 were reported in 61.7% of patients. The most common ≥Grade 3 TEAEs were anaemia and alanine aminotransferase (ALT) increased (both 7.2%). Immune-mediated TEAEs occurred in 17.2% of patients. TEAEs related to either pamiparib or tislelizumab were reported in 82.2% of patients, with nausea the most commonly reported TEAE related to either tislelizumab or pamiparib (in 40.6% of patients; Supplementary Table S4).

**Table 3:** Summary of incidence of treatment-emergent adverse events.

*n* (%)	EOC *BRCA*mut and/or HRD (Arm 1a; *n* = 23)	EOC *BRCA*wt and HRP (Arm 1b; *n* = 23)	TNBC *BRCA*mut and/or HRD (Arm 2; *n* = 19)	mCRPC *BRCA*mut and/or HRD (Arm 3; *n* = 20)	SCLC (Arm 4; *n* = 23)	HER2− G/GEJ cancer (Arm 5; *n* = 20)	Urothelial cancer (Arm 6; *n* = 21)	Pancreatic cancer (Arm 7; *n* = 21)	Exploratory arm^a^ (Arm 8; *n* = 10)	Total (*N* = 180)
Any TEAE	23 (100.0)	23 (100.0)	19 (100.0)	20 (100.0)	23 (100.0)	20 (100.0)	21 (100.0)	21 (100.0)	9 (90.0)	179 (99.4)
≥Grade 3 TEAEs	12 (52.2)	19 (82.6)	8 (42.1)	11 (55.0)	14 (60.9)	14 (70.0)	15 (71.4)	10 (47.6)	8 (80.0)	111 (61.7)
Serious TEAEs	6 (26.1)	16 (69.6)	7 (36.8)	8 (40.0)	14 (60.9)	10 (50.0)	13 (61.9)	8 (38.1)	8 (80.0)	90 (50.0)
Related to either pamiparib or tislelizumab	4 (17.4)	6 (26.1)	2 (10.5)	3 (15.0)	3 (13.0)	4 (20.0)	5 (23.8)	0 (0.0)	2 (20.0)	29 (16.1)
TEAEs leading to death	0 (0.0)	0 (0.0)	0 (0.0)	1 (5.0)	4 (17.4)	0 (0.0)	2 (9.5)	0 (0.0)	1 (10.0)	8 (4.4)
TEAEs leading to discontinuation of pamiparib	5 (21.7)	5 (21.7)	1 (5.3)	0 (0.0)	2 (8.7)	2 (10.0)	7 (33.3)	4 (19.0)	1 (10.0)	27 (15.0)
TEAEs leading to discontinuation of tislelizumab	5 (21.7)	5 (21.7)	1 (5.3)	2 (10.0)	3 (13.0)	3 (15.0)	6 (28.6)	1 (4.8)	1 (10.0)	27 (15.0)
TEAEs leading to discontinuation of both pamiparib and tislelizumab	1 (4.3)	2 (8.7)	0 (0.0)	0 (0.0)	2 (8.7)	1 (5.0)	5 (23.8)	1 (4.8)	1 (10.0)	13 (7.2)
TEAEs leading to reduction of pamiparib dose	2 (8.7)	1 (4.3)	3 (15.8)	0 (0.0)	0 (0.0)	2 (10.0)	0 (0.0)	1 (4.8)	1 (10.0)	10 (5.6)
TEAEs related to either pamiparib or tislelizumab	22 (95.7)	22 (95.7)	17 (89.5)	17 (85.0)	18 (78.3)	15 (75.0)	19 (90.5)	13 (61.9)	5 (50.0)	148 (82.2)
Related to pamiparib	20 (87.0)	22 (95.7)	17 (89.5)	15 (75.0)	18 (78.3)	15 (75.0)	16 (76.2)	13 (61.9)	3 (30.0)	139 (77.2)
Related to tislelizumab	18 (78.3)	18 (78.3)	16 (84.2)	16 (80.0)	15 (65.2)	14 (70.0)	16 (76.2)	13 (61.9)	3 (30.0)	129 (71.7)
Related to pamiparib and tislelizumab	14 (60.9)	17 (73.9)	15 (78.9)	11 (55.0)	10 (43.5)	14 (70.0)	13 (61.9)	13 (61.9)	1 (10.0)	108 (60.0)
Immune-mediated TEAEs	7 (30.4)	6 (26.1)	3 (15.8)	4 (20.0)	4 (17.4)	2 (10.0)	3 (14.3)	0 (0.0)	2 (20.0)	31 (17.2)
Hepatic TEAE^b^	8 (34.8)	7 (30.4)	6 (31.6)	3 (15.0)	5 (21.7)	3 (15.0)	10 (47.6)	2 (9.5)	2 (20.0)	46 (25.6)
≥Grade 3 hepatic TEAEs as the first hepatic TEAE	4 (17.4)	4 (17.4)	0 (0.0)	0 (0.0)	2 (8.7)	2 (10.0)	3 (14.3)	0 (0.0)	0 (0.0)	15 (8.3)

All adverse events were coded using MedDRA version 22.0 and graded according to the NCI CTCAE v4.03.

Data cutoff: 25 September 2020.

*BRCAmut* breast cancer type 1/2 susceptibility gene mutation, *BRCAwt* breast cancer type 1/2 susceptibility gene wildtype, *EOC* epithelial ovarian cancer, *G/GEJ* gastric or gastroesophageal junction, *HER2−* HER2 negative, *HRD* homologous recombination deficiency, *HRP* homologous recombination proficiency, *mCRPC* metastatic castration-resistant prostate cancer, *MedDRA* Medical Dictionary for Regulatory Activities, *NCI CTCAE* National Cancer Institute’s Common Terminology Criteria for Adverse Events, *PD-1* programmed cell death protein 1, *SCLC* small cell lung cancer, *TEAE* treatment-emergent adverse event, *TNBC* triple-negative breast cancer.

^a^Patients with non-ovarian gynaecological cancers (endometrial cancer or cancer of the cervix) and patients with tumours known to be mismatch repair deficient or HRD that are not eligible for inclusion in any other arms of the trial but that may be expected to benefit from the PARP/PD-1 inhibitor combination (see Supplementary Table S1 for the full list of cancer types enrolled in this arm).

^b^Incidence of hepatic TEAEs is reported as defined by the MedDRA according to laboratory terms (alanine aminotransferase increased, aspartate aminotransferase increased, blood bilirubin increased, transaminases increased) or non-laboratory terms (hepatitis, immune-mediated hepatitis, autoimmune hepatitis, hepatic failure). If a patient experienced multiple hepatic TEAEs, only the first hepatic TEAE was counted.

**Table 4:** Incidence of the most common any grade treatment-emergent adverse events.

*n* (%)	EOC *BRCA*mut and/or HRD (Arm 1a; *n* = 23)	EOC *BRCA*wt and HRP (Arm 1b; *n* = 23)	TNBC *BRCA*mut and/or HRD (Arm 2; *n* = 19)	mCRPC *BRCA*mut and/or HRD (Arm 3; *n* = 20)	SCLC (Arm 4; *n* = 23)	HER2− G/GEJ cancer (Arm 5; *n* = 20)	Urothelial cancer (Arm 6; *n* = 21)	Pancreatic cancer (Arm 7; *n* = 21)	Exploratory arm^a^ (Arm 8; *n* = 10)	Total (*N* = 180)
TEAEs occurring in ≥15% of patients in the overall population by preferred term, *n* (%)										
Nausea	18 (78.3)	17 (73.9)	15 (78.9)	8 (40.0)	8 (34.8)	7 (35.0)	12 (57.1)	13 (61.9)	3 (30.0)	101 (56.1)
Fatigue	10 (43.5)	15 (65.2)	12 (63.2)	13 (65.0)	5 (21.7)	11 (55.0)	9 (42.9)	9 (42.9)	5 (50.0)	89 (49.4)
Diarrhoea	10 (43.5)	6 (26.1)	8 (42.1)	4 (20.0)	4 (17.4)	6 (30.0)	6 (28.6)	3 (14.3)	1 (10.0)	48 (26.7)
Constipation	4 (17.4)	8 (34.8)	5 (26.3)	6 (30.0)	3 (13.0)	8 (40.0)	3 (14.3)	6 (28.6)	2 (20.0)	45 (25.0)
Anaemia	5 (21.7)	2 (8.7)	11 (57.9)	5 (25.0)	3 (13.0)	2 (10.0)	5 (23.8)	5 (23.8)	2 (20.0)	40 (22.2)
Back pain	8 (34.8)	4 (17.4)	5 (26.3)	5 (25.0)	5 (21.7)	5 (25.0)	3 (14.3)	3 (14.3)	0 (0.0)	38 (21.1)
Vomiting	6 (26.1)	5 (21.7)	8 (42.1)	3 (15.0)	2 (8.7)	4 (20.0)	4 (19.0)	3 (14.3)	2 (20.0)	37 (20.6)
Decreased appetite	6 (26.1)	4 (17.4)	2 (10.5)	2 (10.0)	8 (34.8)	3 (15.0)	3 (14.3)	5 (23.8)	2 (20.0)	35 (19.4)
Aspartate aminotransferase increased	3 (13.0)	4 (17.4)	3 (15.8)	3 (15.0)	2 (8.7)	2 (10.0)	9 (42.9)	2 (9.5)	2 (20.0)	30 (16.7)
Alanine aminotransferase increased	4 (17.4)	4 (17.4)	3 (15.8)	3 (15.0)	2 (8.7)	2 (10.0)	8 (38.1)	1 (4.8)	2 (20.0)	29 (16.1)
Headache	5 (21.7)	3 (13.0)	5 (26.3)	3 (15.0)	3 (13.0)	3 (15.0)	2 (9.5)	2 (9.5)	1 (10.0)	27 (15.0)

All adverse events were coded using MedDRA version 22.0.

Data cutoff: 25 September 2020.

*BRCAmut* breast cancer type 1/2 susceptibility gene mutation, *BRCAwt*, breast cancer type 1/2 susceptibility gene wildtype, *EOC* epithelial ovarian cancer, *G/GEJ* gastric or gastroesophageal junction, *HER2−* HER2 negative, *HRD* homologous recombination deficiency, *HRP* homologous recombination proficiency, *mCRPC* metastatic castration-resistant prostate cancer, *MedDRA* Medical Dictionary for Regulatory Activities, *PD-1* programmed cell death protein 1, *SCLC* small cell lung cancer, *TEAE* treatment-emergent adverse event, *TNBC* triple-negative breast cancer.

^a^Patients with non-ovarian gynaecological cancers (endometrial cancer or cancer of the cervix) and patients with tumours known to be mismatch repair deficient or HRD that are not eligible for inclusion in any other arms of the trial but that may be expected to benefit from the PARP/PD-1 inhibitor combination (see Supplementary Table S1 for the full list of cancer types enrolled in this arm).

Half of the study patients experienced at least one serious TEAE (50.0%), and 16.1% of patients experienced a serious TEAE related to either pamiparib or tislelizumab. The most commonly reported serious TEAE related to either pamiparib or tislelizumab was immune-mediated hepatitis (3.3%). Eight (4.4%) patients experienced a TEAE leading to death, none of which were considered related to treatment.

In the overall population, 25.6% of patients experienced at least one hepatic TEAE (Table 3). Hepatic TEAEs were defined using investigator selection of MedDRA preferred terms according to laboratory assessed terms (ALT increased, aspartate aminotransferase [AST] increased, blood bilirubin increased, transaminases increased) or non-laboratory assessed terms (hepatitis, immune-mediated hepatitis, autoimmune hepatitis, hepatic failure).

When assessed based on only the first hepatic TEAE experienced by the patient, the most commonly occurring hepatic TEAEs of any grade were ALT increased (12.2% of patients), hepatitis (3.9%), AST increased (2.8%), blood bilirubin increased (2.2%), and immune-mediated hepatitis (2.2%). In total, 8.3% of patients in the overall population experienced a ≥Grade 3 hepatic TEAE as their first hepatic TEAE, with the majority of these patients (eight of the 15 patients) from the EOC arms. The most common first ≥Grade 3 hepatic TEAEs were ALT increased (2.8%), and hepatitis (2.2%).

When assessed based on total incidence regardless of chronology, the most common hepatic TEAEs (with an incidence of ≥10% in the safety analysis set) were AST increased (16.7%) and ALT increased (16.1%). The most common ≥Grade 3 hepatic TEAEs were ALT increased (7.2% of patients), AST increased (4.4%), hepatitis (3.9%), blood bilirubin increased (2.8%), and immune-mediated hepatitis (2.8%). Among those patients who experienced hepatic TEAEs, the first hepatic TEAEs typically began during Cycle 1 or 2 of treatment (median onset: day 40 of treatment [range: day 6–485]). There were no deaths attributed to hepatic TEAEs.

Of the 46 patients who experienced a hepatic TEAE, five patients had ALT or AST >3 × the upper limit of normal (ULN) with a concomitant total bilirubin that was >2 × ULN within a 3-day period. There were four patients who had concomitant alkaline phosphatase (ALP) >2 × ULN within a 3-day period, whilst the remaining patient showed concomitant ALP <2 × ULN within a 3-day period with a serious AE of immune-mediated hepatitis (meeting the criteria for Hy’s law [ALT or AST >3 × ULN; total bilirubin >2 × ULN; no initial findings of cholestasis [no elevation of ALP to >2 × ULN]; and no other reason for the increase in ALT and bilirubin] [35]). The immune-mediated hepatitis was evaluated as related to tislelizumab but not pamiparib by the investigator, and the event resolved following the discontinuation of study drug and steroid treatment.

Overall, TEAEs led to permanent discontinuation of tislelizumab or pamiparib in 15.0% of patients for each drug, and discontinuation of both drugs in 7.2% of patients (Table 3). The most common (incidence >2.0% of patients) TEAEs leading to discontinuation of pamiparib were in the “hepatobiliary disorders” and “investigations” MedDRA system organ classes (4.4% and 2.8% of patients, respectively), and included hepatitis (2.2%) and ALT increased (2.2%). Similarly, the most common TEAEs leading to discontinuation of tislelizumab were also in the “hepatobiliary disorders” and “investigations” system organ classes (6.7% and 3.9% of patients, respectively), and included immune-mediated hepatitis and ALT increased (each 3.3%). In addition, TEAEs led to dose reduction of pamiparib in 5.6% of patients.

### Pharmacokinetics and immunogenicity

The plasma concentrations of pamiparib and tislelizumab indicated that the combination of the two study drugs had no impact on the pharmacokinetic profile of either drug (data not shown). The overall prevalence and incidence of ADAs for tislelizumab were low (3.3% [6/180 patients in the overall population], and 3.2% [5/156 patients in the ADA evaluable population], respectively), and no patients tested positive for neutralising antibodies.

## Discussion

The dose-expansion part of this study was conducted in 180 patients with previously treated locally advanced or metastatic solid tumours, including cohorts of patients with either *BRCA*mut and/or HRD. The combination of pamiparib 40 mg orally twice daily plus tislelizumab 200 mg IV Q3W demonstrated antitumour activity in several tumour types, durable responses in a subset of patients, and a manageable safety profile.

In the overall study population, the ORR was 20.0%, which was consistent with the results previously reported for the dose-escalation part of the study (20.4%) [34], despite variation in tumour type enrolment between the two parts. The highest ORR (47.4%) was seen in the arm enrolling patients with TNBC with *BRCA*mut and/or HRD. Two phase III trials have reported ORRs of a similar magnitude with PARP inhibitor monotherapy in patients with advanced or metastatic TNBC with a germline *BRCA*mut, with ORRs of 61.8% for talazoparib (the EMBRACA trial), and 54.7% for olaparib (the OlympiAD trial) [36, 37]. Furthermore, the OlympiAD trial of olaparib in patients with metastatic TNBC with *BRCA*mut demonstrated a significantly longer PFS compared to standard treatment [37]. However, these trials of PARP inhibitor monotherapy exclusively enrolled patients with *BRCA*mut, which is known to be a predictive biomarker for PARP inhibitor sensitivity [38]. In contrast, among the patients with TNBC in the present study who underwent central germline *BRCA* testing, one-third (5/15) had *BRCA*wt. A *BRCA*wt population may have been less likely to respond to PARP inhibition, although three of these five patients had centrally confirmed HRD (the remaining two were enrolled on the basis of a historical local assessment of a germline *BRCA*mut status that was not confirmed during subsequent central assessment). For context, in the Olaparib Expanded trial in patients with metastatic breast cancer (most with oestrogen receptor-positive HER2− subtype breast cancer and a minority with TNBC), the ORR with olaparib monotherapy in the cohort with a germline mutation in a non-*BRCA*1/2 homologous recombination gene was 33% [39]. In addition, the TNBC patients in the current study were more heavily pre-treated than in the EMBRACA and OlympiAD trials and all had received at least one prior line of systemic therapy in the advanced/metastatic setting. For PD-(L)1 inhibitor monotherapy, low ORRs (typically <10%) have been reported in patients with previously treated metastatic TNBC, albeit with the potential for durable responses in a small subset [40]. The promising response rate for the pamiparib and tislelizumab combination reported herein is of particular interest due to the poor prognosis of patients with metastatic TNBC harbouring *BRCA*mut and/or HRD who have progressed on prior lines of systemic therapy [41, 42].

Previous studies of combinations of PARP inhibitors with PD-(L)1 inhibitors have demonstrated antitumour activity in a variety of tumour types, including in patients with TNBC with *BRCA*mut, although there are limited data from head-to-head clinical trials of the combinations versus their component monotherapies [3, 10, 24, 43–45]. For example, the phase I/II MEDIOLA trial of olaparib plus durvalumab reported an ORR of 58.8%, a median DoR of 12.9 months, and a median PFS of 4.9 months in patients with metastatic TNBC with *BRCA*mut [44]. Meanwhile, the single-arm TOPACIO/KEYNOTE-162 phase II study of niraparib combined with pembrolizumab in advanced/metastatic TNBC reported an ORR of 47% among patients with *BRCA*mut, with a median PFS of 8.3 months [43]. The ORRs in these studies appear comparable to that reported with olaparib alone (54.7%) among patients with metastatic TNBC in the phase III OlympiAD trial [37]; however, cross-trial comparisons should be interpreted cautiously given the potential influence of variations in prior therapy and patient characteristics between studies. In the present study, an ORR of 47.4%, a median DoR of 17.1 months, and a median PFS of 8.4 months were reported with pamiparib plus tislelizumab in patients with TNBC harbouring *BRCA*mut and/or with HRD, consistent with the promising response rate seen in such patients in the prior trials of PARP inhibitors with PD-(L)1 inhibitors [43, 44]. These results support further investigation of such combinations in this setting.

The second highest ORR (30.4%) was seen in the EOC arm harbouring *BRCA*mut and/or with HRD. Previously, a phase I/II study of pamiparib monotherapy reported an ORR of 64.6% in previously treated advanced platinum-sensitive ovarian cancer [24], while in the germline *BRCA*1/2 mutated platinum-sensitive relapsed ovarian cancer cohort of the MEDIOLA trial the ORR was 71.9% with olaparib plus durvalumab [46]. In the recurrent platinum-resistant ovarian cancer cohort (*n* = 53) of the TOPACIO/KEYNOTE-162 phase I/II trial investigating niraparib and pembrolizumab, regardless of *BRCA* status, the ORR was 18%, with three (5%) patients experiencing CR and eight (13%) with PR [47]. However, comparisons between these trials should be interpreted cautiously due to differences in patient populations, most notably the presence of germline *BRCA*mut in all patients in both the pamiparib monotherapy study [24] and in the MEDIOLA trial cohort [46], compared with only 17.4% in the present study. For tislelizumab monotherapy, a phase Ia/b study reported an ORR of 9.8% in previously treated advanced ovarian cancer [48] which, taken together with the results of the present study, suggests that adding pamiparib may enhance antitumour responses. Further investigation into the addition of tislelizumab to pamiparib to improve antitumour activity is needed.

The ORR with pamiparib plus tislelizumab in the urothelial cancer cohort in the present study (28.6%) also appeared encouraging, and was similar to findings reported in the phase III KEYNOTE-045 trial of pembrolizumab as second-line therapy for advanced urothelial cancer (21.1%) [49] and in a phase II trial of tislelizumab in Asian patients with previously treated advanced PD-L1-positive urothelial carcinoma (24.0%) [33]. Although results of the phase II BAYOU trial indicated that addition of olaparib to durvalumab in patients with metastatic urothelial carcinoma did not improve PFS or OS, the combination did appear beneficial in the subgroup of patients with HRD, supporting the continued investigation of PARP inhibitor and PD-(L)1 inhibitor combinations in urothelial cancer [50].

Compared with urothelial cancer, TNBC and EOC harbouring *BRCA*mut and/or with HRD, ORR was lower in the other tumour-specific treatment arms, including in the patients with EOC without *BRCA*mut or HRD. These results suggest that the effect of PARP inhibitors combined with PD-(L)1 inhibitors is affected by both tumour type and mutational signature, and emphasise the need for further research into the combination in carefully defined populations.

It is notable that patients with mCRPC in the present study had a median PFS of 10.4 months (95% CI: 4.3, 16.2) and a median OS of 21.2 months (95% CI: 10.5, NE), which were longer than observed in other treatment arms. For comparison, in a recent phase III trial, median PFS and OS of patients with mCRPC receiving olaparib monotherapy were reported to be 5.8 and 17.5 months, respectively [51]. Accepting the limitation that our mCRPC sample was small (*n*  =  20) and should therefore be interpreted cautiously, these findings suggest that pamiparib in combination with a PD-(L)1 inhibitor, such as tislelizumab, may have improved efficacy in patients with mCRPC and should be further investigated. Similar findings were found in a small study (*n* = 17) of olaparib in combination with the PD-L1 inhibitor durvalumab in mCRPC, which reported a median PFS of 16.1 months among patients with deficiencies in DNA damage repair genes [52].

Overall, the safety profile of the pamiparib plus tislelizumab combination was manageable. The most commonly reported TEAEs were consistent with those reported for the combination in the phase Ia part of the study [34], and more broadly with those reported previously for the individual agents [22, 48, 53]. Although more than half of patients (61.7%) experienced a ≥Grade 3 TEAE, TEAEs led to discontinuation of treatment in a relatively small number of patients (15.0% each for discontinuation of pamiparib and tislelizumab [7.2% discontinued both]). TEAEs leading to dose reduction of pamiparib were also infrequent (5.6% of patients) and lower than reported with other PARP inhibitors (with the caveat of cross-trial comparison) [54–57].

As seen in the dose-escalation part of the study [34], hepatic TEAEs were reported in some patients in this dose-expansion phase. The most commonly reported any grade and ≥Grade 3 hepatic TEAEs (whether reported as the first or subsequent hepatic TEAE) were AST increased (any grade in 16.7% of patients and ≥Grade 3 in 4.4% of patients) and ALT increased (16.1% and 7.2%, respectively), and there were few cases of ≥Grade 3 hepatitis (3.9% of patients), ≥Grade 3 immune-mediated hepatitis (2.8% of patients), and ≥Grade 3 blood bilirubin increased (2.8% of patients). An increased incidence of hepatic TEAEs (particularly increased transaminase levels) has been previously reported with PD-(L)1 inhibitors, such as nivolumab and atezolizumab, as well as PARP inhibitors, such as rucaparib [58, 59]. However, an increased incidence of hepatic TEAEs has not been reported in prior studies of pamiparib or tislelizumab, particularly in terms of a notable elevation in ≥Grade 3 increased transaminase levels [23, 24, 30–33, 48, 53]. There was variation in the incidence of hepatic TEAEs between the tumour-specific study arms in the present study, with greatest incidence in the EOC and urothelial cancer arms. Possible explanations for the higher hepatic TEAEs reported in some arms are prior therapies, predisposition to immune-mediated hepatitis, or liver metastases. Nevertheless, the reported hepatic TEAEs do not appear to compromise the safety and tolerability profile of the combination, with only one of the 46 patients who experienced hepatic TEAEs meeting the criteria for Hy’s law.

The strengths of the study included the assessment of the pamiparib and tislelizumab combination in multiple tumour types, including cohorts with EOC and TNBC, with variability in *BRCA* mutation and HRD status. Potential limitations include the heterogeneous patient population, small sample size, and lack of a head-to-head arms versus PARP inhibitor or PD-1 inhibitor monotherapy, which limits the ability to draw conclusions on the effects of combination therapy versus monotherapy. In addition, the attribution and assignment of the cause of AEs by investigators when patients were on two drugs was subjective and can be challenging. However, the patient numbers and study design are typical of, and appropriate for, a phase I expansion study.

In conclusion, pamiparib in combination with tislelizumab showed evidence of antitumour activity in patients with advanced solid tumours, particularly those with *BRCA*mut and/or HRD tumours, with a manageable safety profile in keeping with the class of agents. This study supports further investigation of this combination strategy, particularly in patients with TNBC with *BRCA*mut and/or HRD.

## Supplementary information

Supplementary Appendix

## Data Availability

On request, and subject to certain criteria, conditions, and exceptions, BeiGene, Ltd., will provide access to individual de-identified participant data from BeiGene-sponsored global interventional clinical studies conducted for medicines (1) for indications that have been approved or (2) in programs that have been terminated. BeiGene will also consider requests for the protocol, data dictionary, and statistical analysis plan. Data requests may be submitted to DataDisclosure@beigene.com.
