# Supplementary material for: Pamiparib in combination with tislelizumab in patients with advanced solid tumours: results from the dose-expansion stage of a multicentre, open-label, phase I trial
Source: Br J Cancer. 2023 Jul 20;129(5):797–810. doi: 10.1038/s41416-023-02349-0 (PMC10449784; doi:10.1038/s41416-023-02349-0)
Supplement: Supplementary file 1 — Supplementary Appendix [file 41416_2023_2349_MOESM1_ESM.docx]

# **Supplementary Appendix**

| Investigators list | Page 2 |
| --- | --- |
| Supplementary Tables | Page 4 |
| Supplementary Figures | Page 11 |
| Study inclusion/exclusion criteria | Page 14 |

# **Investigators list**

| **Country** | **Primary Investigator** | **Primary Centre** |
| --- | --- | --- |
| Australia | Sally Baron-Hay | Northern Cancer Institute |
| Australia | Karen Briscoe | Coffs Harbour Health Campus |
| Australia | Alison Davis | The Canberra Hospital |
| Australia | Sophia Frentzas | Monash Health and Faculty of Medicine |
| Australia | Michael Friedlander | Prince of Wales Hospital |
| Australia | Bo Gao | Blacktown Hospital - Blacktown Cancer and Haematology Centre |
| Australia | Janine Lombard | Calvary Mater Newcastle |
| Australia | Nicole McCarthy | Icon Cancer Care |
| Australia | Tarek Meniawy | Linear Clinical Research Ltd |
| Australia | Linda Mileshkin | Peter MacCallum Cancer Centre - Oncology |
| France | Stephane Champiat | Institut De Cancerologie Gustave Roussy |
| France | Veronique Dieras | Centre Eugene Marquis |
| New Zealand | Michelle Wilson | Auckland City Hospital |
| Spain | Andrés Cervantes Ruipérez | Hospital Clínico Universitario de Valencia |
| Spain | Elena Garralda Cabanas | Hospital Universitario Vall d'Hebrón |
| United States | Carlos Becerra | Texas Oncology - Baylor Charles A. Sammons Cancer Center |
| United States | Jordan Berlin | Vanderbilt University Medical Center (VUMC) |
| United States | Christos Fountzilas | Roswell - Roswell Park Cancer Institute - Medical Oncology |
| United States | Michael Gordon | HonorHealth Research Institute |
| United States | Melissa Johnson | The Sarah Cannon Research Institute - Tennessee Oncology |
| United States | Monica Mita | Cedars Sinai Medical Center - Samuel Oschin Comprehensive C |
| United States | Gligich Oleg | Mount Sinai Comprehensive Cancer Center |
| United States | Mary Peters | Beth Israel Deaconess Medical Center |
| United States | Donald Richards | Texas Oncology- Tyler |
| United States | Alexander Spira | US Oncology - Virginia Cancer Specialists, PC |

# **Supplementary Tables**

**Supplementary Table S1.** Additional baseline characteristics.

|  | **EOC *BRCA*mut and/or HRD**  (Arm 1a; *n* = 23) | **EOC**  ***BRCA*wt  and HRP**  (Arm 1b; *n* = 23) | **TNBC *BRCA*mut and/or HRD**  (Arm 2; *n* = 19) | **mCRPC *BRCA*mut and/or HRD**  (Arm 3; *n* = 20) | **SCLC**  (Arm 4; *n* = 23) | **HER2*–* G/GEJ cancer**  (Arm 5; *n* = 20) | **Urothelial cancer**  (Arm 6; *n* = 21) | **Pancreatic cancer**  (Arm 7; *n* = 21) | **Exploratory arm^a^**  (Arm 8; *n* = 10) | **Total**  (*N* = 180) |
| --- | --- | --- | --- | --- | --- | --- | --- | --- | --- | --- |
| Primary cancer diagnosis, *n* (%) |  |  |  |  |  |  |  |  |  |  |
| Bladder | 0 (0.0) | 0 (0.0) | 0 (0.0) | 0 (0.0) | 0 (0.0) | 0 (0.0) | 12 (57.1) | 0 (0.0) | 0 (0.0) | 12 (6.7) |
| CRPC | 0 (0.0) | 0 (0.0) | 0 (0.0) | 20 (100.0) | 0 (0.0) | 0 (0.0) | 0 (0.0) | 0 (0.0) | 0 (0.0) | 20 (11.1) |
| Fallopian tube | 1 (4.3) | 0 (0.0) | 0 (0.0) | 0 (0.0) | 0 (0.0) | 0 (0.0) | 0 (0.0) | 0 (0.0) | 0 (0.0) | 1 (0.6) |
| Gastric | 0 (0.0) | 0 (0.0) | 0 (0.0) | 0 (0.0) | 0 (0.0) | 7 (35.0) | 0 (0.0) | 0 (0.0) | 0 (0.0) | 7 (3.9) |
| GEJ | 0 (0.0) | 0 (0.0) | 0 (0.0) | 0 (0.0) | 0 (0.0) | 13 (65.0) | 0 (0.0) | 0 (0.0) | 0 (0.0) | 13 (7.2) |
| Metastatic urothelial cancer | 0 (0.0) | 0 (0.0) | 0 (0.0) | 0 (0.0) | 0 (0.0) | 0 (0.0) | 1 (4.8) | 0 (0.0) | 0 (0.0) | 1 (0.6) |
| Ovarian | 21 (91.3) | 22 (95.7) | 0 (0.0) | 0 (0.0) | 0 (0.0) | 0 (0.0) | 0 (0.0) | 0 (0.0) | 0 (0.0) | 43 (23.9) |
| Pancreatic | 0 (0.0) | 0 (0.0) | 0 (0.0) | 0 (0.0) | 0 (0.0) | 0 (0.0) | 0 (0.0) | 21 (100.0) | 0 (0.0) | 21 (11.7) |
| Peritoneal | 1 (4.3) | 1 (4.3) | 0 (0.0) | 0 (0.0) | 0 (0.0) | 0 (0.0) | 0 (0.0) | 0 (0.0) | 0 (0.0) | 2 (1.1) |
| Renal pelvis | 0 (0.0) | 0 (0.0) | 0 (0.0) | 0 (0.0) | 0 (0.0) | 0 (0.0) | 2 (9.5) | 0 (0.0) | 0 (0.0) | 2 (1.1) |
| SCLC | 0 (0.0) | 0 (0.0) | 0 (0.0) | 0 (0.0) | 23 (100.0) | 0 (0.0) | 0 (0.0) | 0 (0.0) | 0 (0.0) | 23 (12.8) |
| TNBC | 0 (0.0) | 0 (0.0) | 19 (100.0) | 0 (0.0) | 0 (0.0) | 0 (0.0) | 0 (0.0) | 0 (0.0) | 1 (10.0) | 20 (11.1) |
| Ureter | 0 (0.0) | 0 (0.0) | 0 (0.0) | 0 (0.0) | 0 (0.0) | 0 (0.0) | 2 (9.5) | 0 (0.0) | 0 (0.0) | 2 (1.1) |
| Urethra | 0 (0.0) | 0 (0.0) | 0 (0.0) | 0 (0.0) | 0 (0.0) | 0 (0.0) | 3 (14.3) | 0 (0.0) | 0 (0.0) | 3 (1.7) |
| Urothelial carcinoma | 0 (0.0) | 0 (0.0) | 0 (0.0) | 0 (0.0) | 0 (0.0) | 0 (0.0) | 1 (4.8) | 0 (0.0) | 0 (0.0) | 1 (0.6) |
| Other | 0 (0.0) | 0 (0.0) | 0 (0.0) | 0 (0.0) | 0 (0.0) | 0 (0.0) | 0 (0.0) | 0 (0.0) | 9 (90.0) | 9 (5.0) |
| Number of metastatic sites, *n* (%) |  |  |  |  |  |  |  |  |  |  |
| 0 | 4 (17.4) | 7 (30.4) | 3 (15.8) | 2 (10.0) | 1 (4.3) | 0 (0.0) | 7 (33.3) | 0 (0.0) | 2 (20.0) | 26 (14.4) |
| 1 | 7 (30.4) | 3 (13.0) | 14 (73.7) | 15 (75.0) | 13 (56.5) | 14 (70.0) | 9 (42.9) | 18 (85.7) | 6 (60.0) | 99 (55.0) |
| 2 | 3 (13.0) | 10 (43.5) | 0 (0.0) | 2 (10.0) | 1 (4.3) | 4 (20.0) | 3 (14.3) | 1 (4.8) | 1 (10.0) | 25 (13.9) |
| ≥3 | 9 (39.1) | 3 (13.0) | 2 (10.5) | 1 (5.0) | 8 (34.8) | 2 (10.0) | 2 (9.5) | 2 (9.5) | 1 (10.0) | 30 (16.7) |
| Location of metastases, *n* (%) |  |  |  |  |  |  |  |  |  |  |
| Bone | 0 (0.0) | 0 (0.0) | 2 (10.5) | 11 (55.0) | 5 (21.7) | 2 (10.0) | 0 (0.0) | 0 (0.0) | 0 (0.0) | 20 (11.1) |
| Liver | 4 (17.4) | 2 (8.7) | 5 (26.3) | 2 (10.0) | 7 (30.4) | 9 (45.0) | 4 (19.0) | 17 (81.0) | 2 (20.0) | 52 (28.9) |
| Spinal cord | 0 (0.0) | 0 (0.0) | 0 (0.0) | 0 (0.0) | 0 (0.0) | 0 (0.0) | 0 (0.0) | 0 (0.0) | 0 (0.0) | 0 (0.0) |
| Brain | 0 (0.0) | 0 (0.0) | 0 (0.0) | 0 (0.0) | 4 (17.4) | 0 (0.0) | 0 (0.0) | 0 (0.0) | 1 (10.0) | 5 (2.8) |
| Lymph nodes | 10 (43.5) | 4 (17.4) | 8 (42.1) | 5 (25.0) | 13 (56.5) | 6 (30.0) | 7 (33.3) | 1 (4.8) | 2 (20.0) | 56 (31.1) |
| Other | 13 (56.5) | 14 (60.9) | 3 (15.8) | 4 (20.0) | 9 (39.1) | 7 (35.0) | 7 (33.3) | 4 (19.0) | 5 (50.0) | 66 (36.7) |

^a^Patients with non-ovarian gynaecological cancers (endometrial cancer or cancer of the cervix) and patients with tumours known to be mismatch repair deficient or HRD that are not eligible for inclusion in any other arms of the trial but that may be expected to benefit from the PARP/PD-1 inhibitor combination were included within the exploratory arm. Other primary diagnoses in this arm included adenocarcinoma of the cervix with lung metastases, adrenocortical carcinoma, cholangio, cholangiocarcinoma, gallbladder, high-grade endometrial stromal sarcoma, neuroendocrine, parotid gland, and thyroid cancer which affected one patient each (10.0%).

Abbreviations: *BRCA*mut, breast cancer type 1/2 susceptibility gene mutation; *BRCA*wt, breast cancer type 1/2 susceptibility gene wildtype; EOC, epithelial ovarian cancer; G/GEJ, gastric or gastroesophageal junction; HER2–, HER2 negative; HRD, homologous recombination deficiency; HRP, homologous recombination proficiency; (m)CRPC, (metastatic) castration-resistant prostate cancer; PD-1, programmed cell death protein 1; SCLC, small cell lung cancer; TNBC, triple-negative breast cancer.

**Supplementary Table S2.** Patient disposition and reasons for study discontinuation.

|  | **EOC *BRCA*mut and/or HRD**  (Arm 1a; *n* = 23) | **EOC**  ***BRCA*wt and HRP**  (Arm 1b; *n* = 23) | **TNBC *BRCA*mut and/or HRD**  (Arm 2; *n* = 19) | **mCRPC *BRCA*mut and/or HRD**  (Arm 3; *n* = 20) | **SCLC**  (Arm 4; *n* = 23) | **HER2*–* G/GEJ cancer**  (Arm 5; *n* = 20) | **Urothelial cancer**  (Arm 6; *n* = 21) | **Pancreatic cancer**  (Arm 7; *n* = 21) | **Exploratory arm^a^**  (Arm 8; *n* = 10) | **Total**  (*N* = 180) |
| --- | --- | --- | --- | --- | --- | --- | --- | --- | --- | --- |
| Discontinued from study | 23 (100.0) | 23 (100.0) | 19 (100.0) | 20 (100.0) | 23 (100.0) | 20 (100.0) | 21 (100.0) | 21 (100.0) | 10 (100.0) | 180 (100.0) |
| Withdrawal by patient | 0 (0.0) | 1 (4.3) | 1 (5.3) | 1 (5.0) | 0 (0.0) | 0 (0.0) | 1 (4.8) | 2 (9.5) | 0 (0.0) | 6 (3.3) |
| Lost to follow-up | 0 (0.0) | 0 (0.0) | 0 (0.0) | 0 (0.0) | 1 (4.3) | 1 (5.0) | 1 (4.8) | 0 (0.0) | 0 (0.0) | 3 (1.7) |
| Death | 13 (56.5) | 14 (60.9) | 10 (52.6) | 9 (45.0) | 21 (91.3) | 17 (85.0) | 15 (71.4) | 18 (85.7) | 7 (70.0) | 124 (68.9) |
| Physician decision | 0 (0.0) | 0 (0.0) | 0 (0.0) | 0 (0.0) | 0 (0.0) | 0 (0.0) | 0 (0.0) | 0 (0.0) | 1 (10.0) | 1 (0.6) |
| Progressive disease | 0 (0.0) | 0 (0.0) | 0 (0.0) | 0 (0.0) | 0 (0.0) | 0 (0.0) | 0 (0.0) | 0 (0.0) | 0 (0.0) | 0 (0.0) |
| Study terminated by sponsor | 0 (0.0) | 0 (0.0) | 0 (0.0) | 0 (0.0) | 0 (0.0) | 0 (0.0) | 0 (0.0) | 0 (0.0) | 1 (10.0) | 1 (0.6) |
| Other reasons | 10 (43.5) | 8 (34.8) | 8 (42.1) | 10 (50.0) | 1 (4.3) | 2 (10.0) | 4 (19.0) | 1 (4.8) | 1 (10.0) | 45 (25.0) |

Note: Data cutoff: 25 September 2020.

^a^Patients with non-ovarian gynaecological cancers (endometrial cancer or cancer of the cervix) and patients with tumours known to be mismatch repair deficient or HRD) that are not eligible for inclusion in any other arms of the trial but that may be expected to benefit from the PARP/PD-1 inhibitor combination (see Supplementary Table S1 for the full list of cancer types enrolled in this arm).

Abbreviations: *BRCA*mut, breast cancer type 1/2 susceptibility gene mutation; *BRCA*wt, breast cancer type 1/2 susceptibility gene wildtype; EOC, epithelial ovarian cancer; G/GEJ, gastric or gastroesophageal junction; HER2–, HER2 negative; HRD, homologous recombination deficiency; HRP, homologous recombination proficiency; mCRPC, metastatic castration-resistant prostate cancer; PD-1, programmed cell death protein 1; SCLC, small cell lung cancer; TNBC, triple-negative breast cancer.

**Supplementary Table S3.** Progression free survival and overall survival.

|  | **EOC *BRCA*mut and/or HRD**  (Arm 1a; *n* = 23) | **EOC**  ***BRCA*wt and HRP**  (Arm 1b; *n* = 23) | **TNBC *BRCA*mut and/or HRD**  (Arm 2; *n* = 19) | **mCRPC *BRCA*mut and/or HRD**  (Arm 3; *n* = 20) | **SCLC**  (Arm 4; *n* = 23) | **HER2*–* G/GEJ cancer**  (Arm 5; *n* = 20) | **Urothelial cancer**  (Arm 6; *n* = 21) | **Pancreatic cancer**  (Arm 7; *n* = 21) | **Exploratory arm^a^**  (Arm 8; *n* = 10) | **Total**  (*N* = 180) |
| --- | --- | --- | --- | --- | --- | --- | --- | --- | --- | --- |
| PFS rate, % (95% CI) |  |  |  |  |  |  |  |  |  |  |
| 3 months | 95.7 (72.9, 99.4) | 50.0 (27.1, 69.2) | 93.8 (63.2, 99.1) | 81.9 (53.8, 93.8) | 29.8 (12.9, 48.9) | 33.3 (13.2, 55.0) | 54.2 (30.3, 73.0) | 10.0 (1.7, 27.2) | 44.4  (13.6, 71.9) | 54.7 (46.8, 62.0) |
| 6 months | 67.1 (43.1, 82.8) | 33.8 (14.4, 54.4) | 62.5 (34.9, 81.1) | 60.1 (31.3, 80.0) | 19.9 (6.5, 38.5) | 26.6 (8.8, 48.5) | 37.9 (17.3, 58.5) | 5.0 (0.3, 20.5) | 33.3 (7.8, 62.3) | 38.3 (30.7, 45.8) |
| 9 months | 36.1 (16.4, 56.4) | 16.9 (4.3, 36.6) | 48.6  (22.9, 70.3) | 51.5  (23.5, 73.8) | 9.9  (1.7, 26.8) | 13.3 (2.3, 34.1) | 17.3 (3.6, 39.7) | NR (NE, NE) | 33.3 (7.8, 62.3) | 24.4 (17.8, 31.6) |
| 12 months | 25.8 (9.5, 45.9) | 5.6 (0.4, 22.6) | 41.7 (17.7, 64.3) | 34.3 (11.2, 59.3) | 0.0 (NE, NE) | 13.3 (2.3, 34.1) | 17.3 (3.6, 39.7) | NR (NE, NE) | 33.3 (7.8, 62.3) | 17.3 (11.6, 24.0) |
| OS rate, % (95% CI) |  |  |  |  |  |  |  |  |  |  |
| 3 months | 100.0 (NE, NE) | 91.3 (69.5, 97.8) | 100.0 (NE, NE) | 100.0 (NE, NE) | 73.9 (50.9, 87.3) | 80.0 (55.1, 92.0) | 95.2 (70.7, 99.3) | 71.4 (47.2, 86.0) | 60.0 (25.3, 82.7) | 87.2 (81.3, 91.3) |
| 6 months | 91.3 (69.5, 97.8) | 77.8 (54.6, 90.1) | 88.9 (62.4, 97.1) | 89.5 (64.1, 97.3) | 56.5 (34.3, 73.8) | 55.0 (31.3, 73.5) | 60.2 (35.9, 77.7) | 19.0 (5.9, 37.7) | 40.0 (12.3, 67.0) | 65.6 (58.1, 72.1) |
| 9 months | 82.6 (60.1, 93.1) | 59.5 (36.5, 76.5) | 88.9 (62.4, 97.1) | 84.2 (58.7, 94.6) | 39.1 (19.9, 58.0) | 40.0 (19.3, 60.0) | 44.6 (22.5, 64.5) | 9.5 (1.6, 26.1) | 40.0 (12.3, 67.0) | 54.8 (47.1, 61.8) |
| 12 months | 78.3 (55.4, 90.3) | 59.5 (36.5, 76.5) | 66.2 (39.6, 83.2) | 68.4 (42.8, 84.4) | 29.3 (12.5, 48.5) | 35.0 (15.7, 55.2) | 33.4 (14.1, 54.1) | 4.8 (0.3, 19.7) | 30.0 (7.1, 57.8) | 46.0 (38.5, 53.2) |
| 15 months | 64.7 (41.6, 80.6) | 59.5 (36.5, 76.5) | 60.2 (34.0, 78.7) | 68.4 (42.8, 84.4) | 29.3 (12.5, 48.5) | 20.0 (6.2, 39.3) | 33.4 (14.1, 54.1) | 4.8 (0.3, 19.7) | 30.0 (7.1, 57.8) | 41.7 (34.3, 48.9) |

Note: PFS and OS rates with 95% CI were calculated using Kaplan–Meier estimate and Greenwood Formula. Data cutoff: 25 September 2020.

^a^Patients with non-ovarian gynaecological cancers (endometrial cancer or cancer of the cervix) and patients with tumours known to be mismatch repair deficient or HRD) that are not eligible for inclusion in any other arms of the trial but that may be expected to benefit from the PARP/PD-1 inhibitor combination (see Supplementary Table S1 for the full list of cancer types enrolled in this arm).

Abbreviations: *BRCA*mut, breast cancer type 1/2 susceptibility gene mutation; *BRCA*wt, breast cancer type 1/2 susceptibility gene wildtype; CI, confidence interval; EOC, epithelial ovarian cancer; G/GEJ, gastric or gastroesophageal junction; HER2–, HER2 negative; HRD, homologous recombination deficiency; HRP, homologous recombination proficiency; mCRPC, metastatic castration-resistant prostate cancer; NE, not estimable; NR, not reached; OS, overall survival; PD-1, programmed cell death protein 1; PFS, progression-free survival; SCLC, small cell lung cancer; TNBC, triple-negative breast cancer.

**Supplementary Table S4.** Incidence of treatment-emergent adverse events related to either pamiparib or tislelizumab.

| ***n* (%)** | **EOC *BRCA*mut and/or HRD**  (Arm 1a; *n* = 23) | **EOC *BRCA*wt and HRP**  (Arm 1b; *n* = 23) | **TNBC *BRCA*mut and/or HRD**  (Arm 2; *n* = 19) | **mCRPC *BRCA*mut and/or HRD**  (Arm 3; *n* = 20) | **SCLC**  (Arm 4; *n* = 23) | **HER2–G/GEJ cancer**  (Arm 5; *n* = 20) | **Urothelial cancer**  (Arm 6; *n* = 21) | **Pancreatic cancer** (Arm 7; *n* = 21) | **Exploratory arm^a^**  (Arm 8; *n* = 10) | **Total**  (*N* = 180) |
| --- | --- | --- | --- | --- | --- | --- | --- | --- | --- | --- |
| Treatment-related TEAEs occurring in ≥10% of patients in the overall population by preferred term, *n* (%) |  |  |  |  |  |  |  |  |  |  |
| Nausea | 15 (65.2) | 15 (65.2) | 11 (57.9) | 4 (20.0 | 7 (30.4) | 4 (20.0) | 8 (38.1) | 7 (33.3) | 2 (20.0) | 73 (40.6) |
| Fatigue | 9 (39.1) | 13 (56.5) | 7 (36.8) | 8 (40.0) | 4 (17.4) | 8 (40.0) | 5 (23.8) | 7 (33.3) | 1 (10.0) | 62 (34.4) |
| Diarrhoea | 5 (21.7) | 3 (13.0) | 5 (26.3) | 2 (10.0) | 2 (8.7) | 4 (20.0) | 5 (23.8) | 1 (4.8) | 1 (10.0) | 28 (15.6) |
| Anaemia | 4 (17.4) | 2 (8.7) | 6 (31.6) | 4 (20.0) | 1 (4.3) | 2 (10.0) | 4 (19.0) | 0 (0.0) | 0 (0.0) | 23 (12.8) |
| Vomiting | 4 (17.4) | 3 (13.0) | 4 (21.1) | 2 (10.0) | 1 (4.3) | 2 (10.0) | 2 (9.5) | 1 (4.8) | 1 (10.0) | 20 (11.1) |
| Aspartate aminotransferase increased | 2 (8.7) | 2 (8.7) | 1 (5.3) | 3 (15.0) | 1 (4.3) | 2 (10.0) | 7 (33.3) | 2 (9.5) | 1 (10.0) | 21 (11.7) |
| Alanine aminotransferase increased | 2 (8.7) | 3 (13.0) | 2 (10.5) | 3 (15.0) | 1 (4.3) | 2 (10.0) | 6 (28.6) | 1 (4.8) | 1 (10.0) | 21 (11.7) |

Note: All adverse events were coded using MedDRA version 22.0. Data cutoff: 25 September 2020.

^a^Patients with non-ovarian gynaecological cancers (endometrial cancer or cancer of the cervix) and patients with tumours known to be mismatch repair deficient or HRD) that are not eligible for inclusion in any other arms of the trial but that may be expected to benefit from the PARP/PD-1 inhibitor combination (see Supplementary Table S1 for the full list of cancer types enrolled in this arm).

Abbreviations: *BRCA*mut, breast cancer type 1/2 susceptibility gene mutation; *BRCA*wt, breast cancer type 1/2 susceptibility gene wildtype; EOC, epithelial ovarian cancer; G/GEJ, gastric or gastroesophageal junction; HER2–, HER2 negative; HRD, homologous recombination deficiency; HRP, homologous recombination proficiency; mCRPC, metastatic castration-resistant prostate cancer; MedDRA, Medical Dictionary for Regulatory Activities; PD-1, programmed cell death protein 1; SCLC, small cell lung cancer; TEAE, treatment-emergent adverse event; TNBC, triple-negative breast cancer.

# **Supplementary Figures**

**Supplementary Figure S1.** Study design.


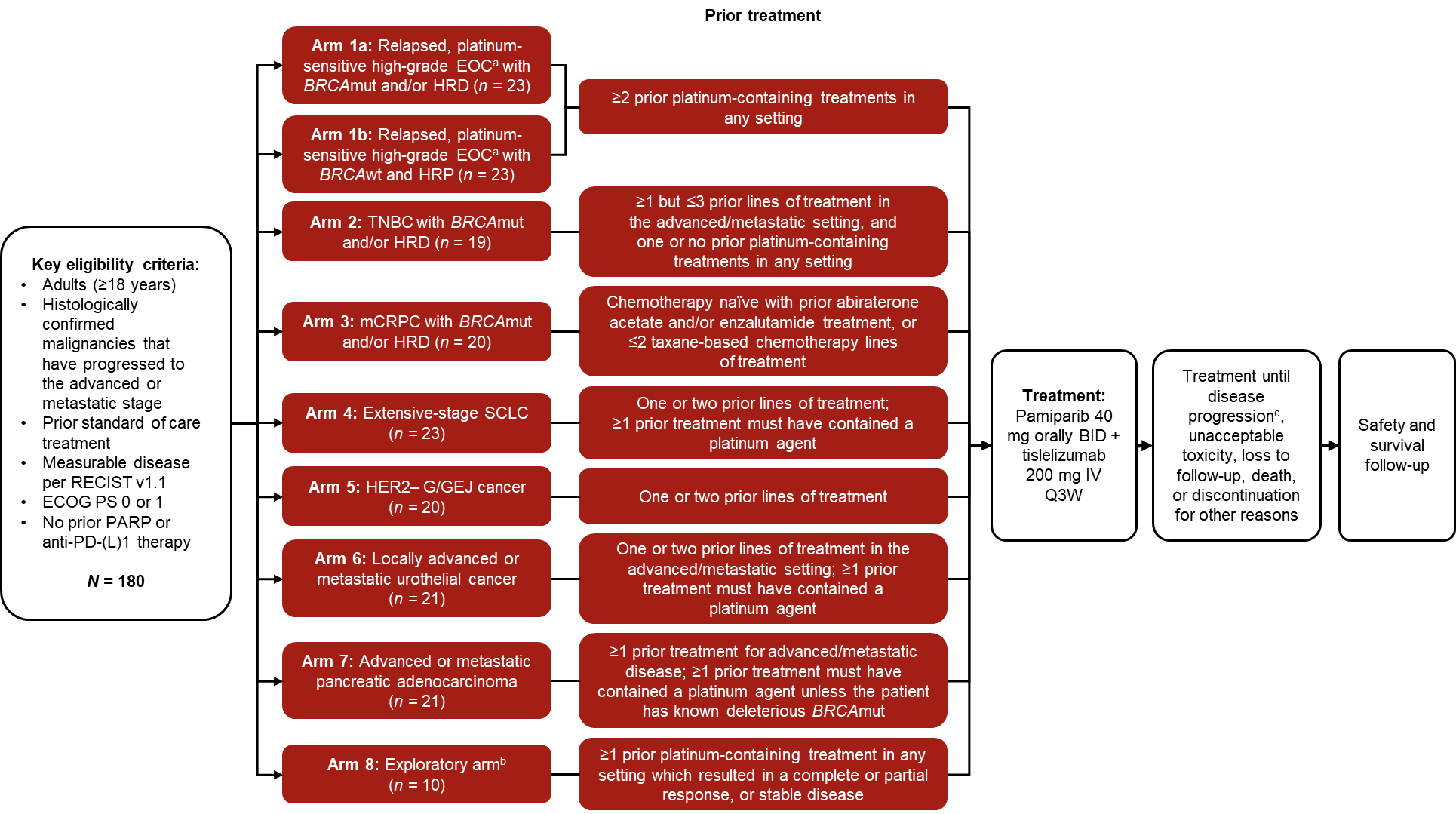


^a^Includes epithelial, non-mucinous, ovarian cancer, fallopian tube, or primary peritoneal cancer.

^b^Patients with advanced or metastatic recurrent non-ovarian gynaecological cancers (endometrial cancer or cancer of the cervix) and patients with tumours known to be either mismatch repair deficient or HRD that were not eligible for inclusion in any other arms of the trial but that may be expected to benefit from the combination of a PARP inhibitor and a PD-1 inhibitor (see Supplementary Table S1 for the full list of cancer types enrolled in this arm).

^c^Continued treatment beyond progression was permitted if “pseudo-progression” was suspected by the investigator, provided the following criteria were met: investigator-assessed clinical benefit; toleration of study drug by the patient and agreement to continue treatment; stable ECOG PS; absence of rapid disease progression or of progressive tumours at critical anatomical sites requiring urgent intervention; and reconsent to continue study treatment.

BID, twice a day; *BRCA*mut, breast cancer type 1/2 susceptibility gene; *BRCA*wt, breast cancer type 1/2 susceptibility gene wildtype; ECOG PS, Eastern Cooperative Oncology Group performance status; EOC, epithelial ovarian cancer; G/GEJ, gastric or gastroesophageal junction; HER2–, HER2 negative; HRD, homologous recombination deficiency; HRP, homologous recombination proficiency; IV, intravenously; mCRPC, metastatic castration-resistant prostate cancer; PD-1, programmed cell death protein 1; PD-L1, programmed death-ligand 1; Q3W, every 3 weeks; RECIST v1.1, Response Evaluation Criteria in Solid Tumours version 1.1; SCLC, small cell lung cancer; TNBC, triple-negative breast cancer.

**Supplementary Figure S2.** Progression-free survival (a) and overall survival (b) in the TNBC *BRCA*mut and/or HRD arm (Arm 2).*


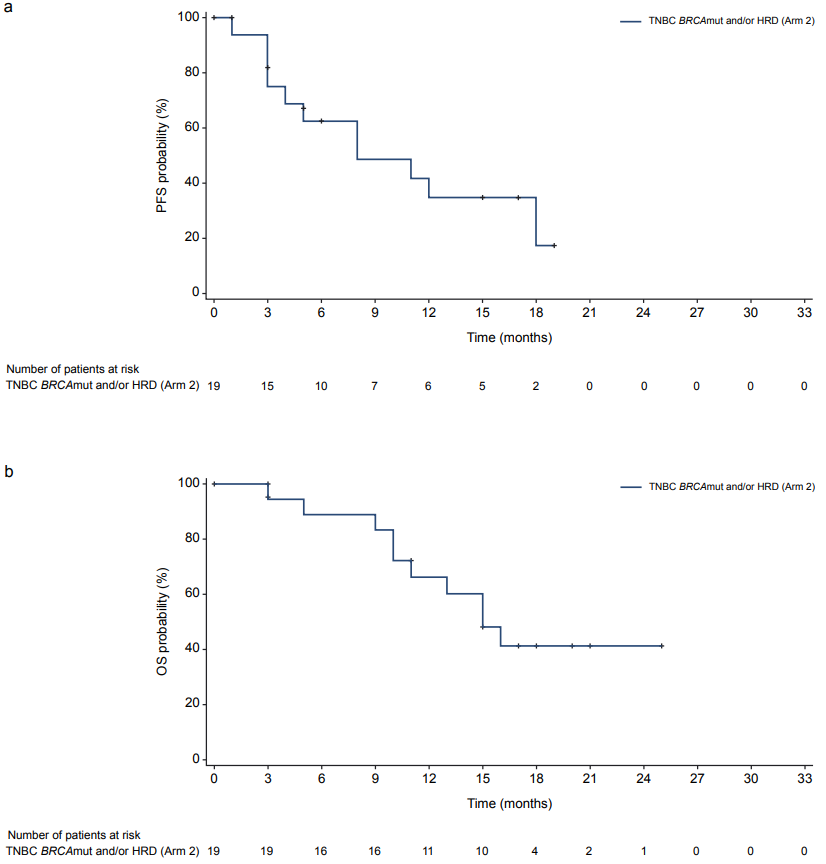


***Data for other arms is presented in Figure 2 within the main manuscript.
Data cutoff: 25 September 2020.  *BRCA*mut, breast cancer type 1/2 susceptibility gene mutation; HRD, homologous recombination deficiency; OS, overall survival; PFS, progression-free survival; TNBC, triple-negative breast cancer.

# **Study inclusion/exclusion criteria**

Inclusion criteria

To be eligible to participate in this study, a patient was required to meet all of the following criteria:

1. Patients have voluntarily agreed to participate by giving written informed consent.

2. Patients must have received standard of care in the primary treatment of their disease.

3. Patients who have the below specified histologically confirmed malignancies that have progressed to the advanced or metastatic stage.

In part B, the patients recruited to one of the eight expansion arms must have advanced solid tumours of the following types:

Arm 1: Patients with relapsed, platinum-sensitive high grade epithelial, non-mucinous, ovarian cancer, fallopian tube, or primary peritoneal cancer (EOC) must meet the following criteria:

i. Patients must have at received least 2 prior platinum-containing treatments in any treatment setting.

- Note: Patients may have received additional therapy after the last platinum-containing line of treatment if the other eligibility criteria are met.

ii. Patients must have platinum-sensitive recurrent disease and must not have progressed (by Response Evaluation Criteria in Solid Tumours [RECIST] v1.1 criteria) within 6 months of the completion of the last platinum-containing line of treatment.

- Note: Patients may receive additional non-platinum based chemotherapy for recurrence after the prior platinum-containing line of treatment if the criteria for platinum-sensitivity are met.

iii. Arm 1a: Known deleterious or suspected deleterious germline or somatic *BRCA*1/2 mutations or with homologous recombination deficiency (HRD).

- - - If HRD or *BRCA*1/2 mutation status from archival tissue is unknown or has not been previously evaluated, then the archival tissue must undergo tissue screening using a validated diagnostic test to determine eligibility. If the diagnostic test result is *BRCA*1/2 or HRD the patient will be eligible for enrolment in Arm 1a.

iv. Arm 1b: Without known germline or somatic *BRCA*1/2 mutations and without HRD mutation.

Arm 2: Patients with triple-negative breast cancer must meet the following criteria:

i. Patients with 0–1 prior platinum-containing treatment in any treatment setting.

- Note: Patients could have received additional therapy after the last platinum-containing line of treatment if the other eligibility criteria are met.

ii. Patients who have received at least 1 but no more than 3 prior lines of treatment in the advanced or metastatic setting.

iii. Known deleterious or suspected deleterious germline or somatic *BRCA*1/2 mutations or with documented HRD.

If HRD or *BRCA*1/2 mutation status from archival tissue is unknown or has not been previously evaluated, then the archival tissue must undergo tissue screening using a validated diagnostic test to determine eligibility. If the diagnostic test result is HRD, then the patient will be eligible for enrolment in Arm 2.

- If archival tissue is not available and the patient submits a fresh tumour biopsy, then the diagnostic test needs to demonstrate somatic *BRCA*1/2 mutation or HRD positivity.

Arm 3: Patients with metastatic castration-resistant prostate cancer, including but not limited to mutations in HR pathways and/or defined by HRD algorithms, and must meet the following criteria:

i. The patient may be either chemotherapy-naïve, but must have received prior abiraterone acetate and/or enzalutamide treatment, or have previously had no more than 2 taxane-based chemotherapy lines of treatment including docetaxel and carbazitaxel. If docetaxel is used more than once, this will be considered as 1 line of treatment.

ii. At least 2 weeks since the completion of prior flutamide, bicalutamide, and nilutimide, or enzalutamide and abiraterone treatment.

iii. Documented prostate cancer with one of the following:

- Surgically or medically castrated. The testosterone levels do not need to be checked if the patient has undergone surgical castration for >4 months. Patients receiving chemical castration should have testosterone levels checked at baseline and confirmed to be in the castrate levels (<0.5 ng/mL or 1.735 nM). In all cases the luteinizing hormone-releasing hormone antagonist/agonist is to be continued in these patients.
- Patients with only non-measurable bone lesions must have disease progression based on Prostate Cancer Clinical Trials Working Group 3 criteria with 2 or more new lesions or have prostate-specific antigen progression before enrolment.

iv. Known deleterious or suspected deleterious germline or somatic *BRCA*1/2 mutations or with documented HRD.

- If HRD or *BRCA*1/2 mutation status from archival tissue is unknown or has not been previously evaluated, then the archival tissue must undergo tissue screening using a validated diagnostic test to determine eligibility. If the diagnostic test result is HRD, then the patient will be eligible for enrolment in Arm 3.
- If archival tissue is not available and the patient submits a fresh tumour biopsy, then the diagnostic test needs to demonstrate somatic *BRCA*1/2 mutation or HRD positivity.

Arm 4: Patients with extensive-stage disease SCLC must meet the following criterion:

i. Patients received at least 1 but no more than 2 prior lines of treatment.

ii. At least 1 prior treatment for must have contained a platinum agent.

Arm 5: Patients with HER2-negative gastric or gastroesophageal junction cancer must meet the following criteria:

i. Received at least 1 but no more than 2 prior lines of treatment.

Arm 6: Patients with locally advanced or metastatic urothelial (muscle-invasive bladder, ureter, urethra, or renal pelvis) cancer must meet the following criteria:

i. At least 1 but no more than 2 prior lines of treatment in the advanced or metastatic disease setting.

ii. At least 1 prior treatment must have contained a platinum agent.

Arm 7: Patients with advanced or metastatic pancreatic adenocarcinoma must meet the following criteria:

i. At least 1 prior treatment for advanced or metastatic disease.

ii. At least 1 prior treatment must have contained a platinum agent.

iii. Patients with known deleterious germline or somatic *BRCA*1/2 mutations can be considered for the study even if platinum-naïve.

Arm 8: Patients with advanced or metastatic recurrent non-ovarian gynaecological cancers (endometrial cancer, cancer of the cervix, and patients with tumours known to be mismatch repair [MMR] deficient or HRD) must meet the following criteria:

i. Patients with a complete response, partial response, or stable disease from at least 1 prior platinum-containing treatment in any treatment setting.

ii. The Sponsor medical monitor will approve tumour types for Arm 8 prior to screening.

Note: Excluded tumour types include patients with bone or soft tissue sarcoma; central nervous system (CNS) malignancies; colorectal cancer (except microsatellite instability-high colorectal cancer is permitted); cutaneous or ocular melanoma; hematologic malignancies; HER2-negative breast cancer without *BRCA* mutation; mesothelioma, papillary, follicular, medullary, or Hürthle cell thyroid cancer; unknown primary malignancy.

4. Patients who were treated with chemotherapy or any investigational therapies, if eligible, must have been completed at least 4 weeks or at least 5 half-lives (whichever is longer, but no less than 3 weeks) before the study drug administration, and all adverse events (AEs) have either returned to baseline or stabilised.

5. At least 2 weeks from palliative radiotherapy.

6. Patients must have archival tumour tissue or agree to a tumour biopsy for biomarkers analysis unless previously discussed with sponsor’s medical monitor or its designee (fresh tumour biopsies are recommended at baseline in patients with readily accessible tumour lesions who can safely undergo the procedure and who consent to the biopsies). All patients enrolled in part B must also agree to provide fresh blood sample at the baseline for the evaluation of *BRCA*mut and/or confirmation of prior *BRCA* results or other homologous recombination deficiency mutations even if it was previously tested.

7. Patients must have measurable disease as defined in RECIST v1.1. Patients with metastatic castration-resistant prostate cancer and epithelial, non-mucinous, ovarian cancer, fallopian tube, or primary peritoneal cancer may use separate disease-specific criteria.

8. Patients must be a male or female ≥18 years of age on the day of signing informed consent.

9. Patients must have an Eastern Cooperative Oncology Group (ECOG) performance status (PS) ≤1.

10. Patients must have a life expectancy ≥12 weeks.

11. Patient must have adequate organ function as indicated by the following laboratory values independent of transfusion within 2 weeks:

a) Absolute neutrophil count ≥1500/mL.

b) Platelets ≥100,000/mL.

c) Haemoglobin ≥9 g/dL or ≥5.6 mmol/L.

d) Estimated glomerular filtration rate ≥30 mL/min/1.73 m^2^ by Chronic Kidney Disease Epidemiology Collaboration equation.

e) Serum total bilirubin ≤1.5 × upper limit of normal (ULN) (On fractionation ≤90% of total bilirubin should be unconjugated. Total bilirubin must be <4 × ULN for patients with Gilbert’s syndrome).

f) Aspartate aminotransferase (serum glutamic oxaloacetic transaminase) and alanine aminotransferase (serum glutamic pyruvic transaminase) ≤3 × ULN.

g) International normalised ratio ≤1.5 × ULN (≤2.5 × ULN if on anticoagulants).

12. Females of childbearing potential must be willing to use a highly effective method of birth control for the duration of the study, and for at least 6 months after the last dose of investigational drug, and have a negative serum pregnancy test within 7 days of the first dose of study drug(s).

13. Non-sterile males and their female partners must be willing to use a highly effective method of birth control for the duration of the study and for at least 6 months after the last dose of investigational drug. Non-sterile males must avoid sperm donation for the duration of the study and for at least 6 months after last study drug.

14. Female patient must agree not to breastfeed starting at screening and throughout the study period, and for 6 months after the final study drug administration.

Exclusion criteria

Patients will not be enrolled in the study for any of the following reasons:

1. Patients with ovarian cancer who have platinum-resistant/refractory disease, defined as progressive disease at the first RECIST v1.1 tumour assessment while receiving platinum-containing chemotherapy.

2. Patient has history of severe hypersensitivity reactions to other monoclonal antibodies.

3. Any major surgery within 28 days before first dose of study drugs.

4. Prior allogeneic stem cell transplantation or organ transplantation.

5. Patients with toxicities (as a result of prior anticancer therapy) which have not recovered to baseline or stabilised, except for AEs not considered a likely safety risk (e.g. alopecia, neuropathy, and specific laboratory abnormalities).

6. Concurrent participation in another clinical trial.

7. Prior malignancy within the previous 2 years except for locally curable non-melanoma dermatologic cancers that have been treated with curative intent and are at very low risk for recurrence, such as basal or squamous cell skin cancer, or carcinoma in situ of the skin, cervix, breast, bladder, or prostate.

8. Symptomatic CNS metastasis or leptomeningeal disease.

Note: Baseline MRI of the brain and spinal cord is required for SCLC patients enrolled in Arm 4.

Note: Patients with previously treated CNS metastatic disease are eligible for any arm if CNS metastatic disease is asymptomatic, clinically stable, and does not require corticosteroids or anticonvulsants within a minimum of 4 weeks of enrolment.

9. Prior therapies targeting PD-1, programmed death-ligand 1 (PD-L1), or PARP.

10. Active autoimmune diseases or history of autoimmune diseases that may relapse.

Note: Patients with the following diseases are not excluded and may proceed to further screening:

a) Controlled Type I diabetes.

b) Hypothyroidism managed with no treatment other than with hormone replacement therapy.

c) Controlled celiac disease.

d) Skin diseases not requiring systemic treatment (e.g. vitiligo, psoriasis, alopecia).

e) Any other disease that is not expected to recur in the absence of external triggering factors.

11. Any condition that required systemic treatment with either corticosteroids (>10 mg daily of prednisone or equivalent) or other immunosuppressive medication within 2 weeks of the study drug administration.

Note: Patients who are currently or have previously been on any of the following steroid regimens are not excluded:

a) Adrenal replacement steroid (dose ≤10 mg daily of prednisone or equivalent).

b) Topical, ocular, intra-articular, intranasal, or inhalational corticosteroid with minimal systemic absorption.

c) Short course (≤7 days) of corticosteroid prescribed prophylactically (e.g. for contrast dye allergy) or for the treatment of a non-autoimmune condition (e.g. delayed-type hypersensitivity reaction caused by contact allergen).

12. With severe chronic or active infections requiring systemic antibacterial, antifungal or antiviral therapy, including tuberculosis infection, etc.

13. History of interstitial lung disease, non-infectious pneumonitis or uncontrolled systemic diseases, including diabetes, hypertension, pulmonary fibrosis, acute lung diseases, etc.

14. History of non-viral hepatitis or cirrhosis.

15. Positive human immunodeficiency virus status.

16. A known history of hepatitis B virus, or hepatitis C virus infection.

17. History of alcohol abuse.

18. Underlying medical conditions or alcohol or drug abuse or dependence that, in the investigator’s opinion, will be unfavourable for the administration of study drug or affect the explanation of drug toxicity or adverse events; or insufficient compliance during the study according to investigator’s judgement.

19. Inability to swallow oral medications (capsules and tablets) without chewing, breaking, crushing, opening, or otherwise altering the product formulation. Patients should not have gastrointestinal illnesses that would preclude the absorption of pamiparib, which is an oral agent.

20. Has been administered a live vaccine within 4 weeks (28 days) of initiation of study therapy. Patients are eligible if 28 days have elapsed since receipt of vaccine and initiation of study treatment. (Note: Seasonal vaccines for influenza are generally inactivated vaccines and are allowed. Intranasal vaccines are live vaccines; and are not allowed).

21. Any of the following cardiovascular criteria:

a) Current evidence of cardiac ischaemia.

b) Current symptomatic pulmonary embolism.

c) Acute myocardial infarction ≤6 months prior to Day 1.

d) Heart failure of New York Heart Association Classification III or IV ≤6 months prior to Day 1.

e) Grade ≥2 ventricular arrhythmia ≤6 months prior to Day 1.

f) History of cerebrovascular accident within 6 months before first dose of study drugs.

22. Use or have anticipated need for food or drugs known to be strong or moderate cytochrome P450 (CYP)3A inhibitors or strong CYP3A inducers ≤10 days (or ≤5 half-lives, whichever is shorter) prior to Day 1.
